# Genetic, epigenetic and environmental factors in diverticular disease: systematic review

**DOI:** 10.1093/bjsopen/zrae032

**Published:** 2024-06-04

**Authors:** Hannah N Humphrey, Pauline Sibley, Eleanor T Walker, Deborah S Keller, Francesco Pata, Dale Vimalachandran, Ian R Daniels, Frank D McDermott

**Affiliations:** Department of Colorectal Surgery, Royal Devon University Healthcare Foundation Trust, Exeter, UK; Department of Colorectal Surgery, Royal Devon University Healthcare Foundation Trust, Exeter, UK; Department of Colorectal Surgery, Royal Devon University Healthcare Foundation Trust, Exeter, UK; Department of Surgery, Lankenau Medical Center and Lankenau Institute for Medical Research, Wynnewood, Pennsylvania, USA; Department of Pharmacy, Health and Nutritional Sciences, University of Calabria, Rende, Italy; Department of Molecular & Cancer Medicine, Institute of Cancer Medicine, University of Liverpool, Liverpool, UK; Department of Colorectal Surgery, Countess of Chester Hospital NHS Foundation Trust, Chester, UK; Department of Colorectal Surgery, Royal Devon University Healthcare Foundation Trust, Exeter, UK; Department of Colorectal Surgery, Royal Devon University Healthcare Foundation Trust, Exeter, UK

## Abstract

**Background:**

Diverticulosis is a normal anatomical variant of the colon present in more than 70% of the westernized population over the age of 80. Approximately 3% will develop diverticulitis in their lifetime. Many patients present emergently, suffer high morbidity rates and require substantial healthcare resources. Diverticulosis is the most common finding at colonoscopy and has the potential for causing a significant morbidity rate and burden on healthcare. There is a need to better understand the aetiology and pathogenesis of diverticular disease. Research suggests a genetic susceptibility of 40–50% in the formation of diverticular disease. The aim of this review is to present the hypothesized functional effects of the identified gene loci and environmental factors.

**Methods:**

A systematic literature review was performed using PubMed, MEDLINE and Embase. Medical subject headings terms used were: ‘diverticular disease, diverticulosis, diverticulitis, genomics, genetics and epigenetics’. A review of grey literature identified environmental factors.

**Results:**

Of 995 articles identified, 59 articles met the inclusion criteria. Age, obesity and smoking are strongly associated environmental risk factors. Intrinsic factors of the colonic wall are associated with the presence of diverticula. Genetic pathways of interest and environmental risk factors were identified. The *COLQ, FAM155A, PHGR1, ARHGAP15, S100A10*, and *TNFSF15* genes are the strongest candidates for further research.

**Conclusion:**

There is increasing evidence to support the role of genomics in the spectrum of diverticular disease. Genomic, epigenetic and omic research with demographic context will help improve the understanding and management of this complex disease.

## Teaser

There is a need to better understand the aetiology and pathogenesis of diverticular disease. Research suggests a genetic susceptibility of 40–50% in the formation of diverticular disease, supported by further genome-wide association studies. The aim of this review is to present the hypothesized functional effects of the identified gene loci.

## Introduction

Diverticular disease (DD) is a heterogenous disease of unknown molecular pathophysiological origin with increasing incidence and lack of an international unifying clinical management strategy. DD covers the spectrum of asymptomatic diverticulosis to complicated diverticulitis. Diverticulosis is a common anatomical variant found at colonoscopy in 15% of patients in their fifth decade of life and gradually increasing to 70% by the eighth decade^1–3^. Approximately 20% of patients with diverticulosis will develop symptomatic DD of which 15% will go on to develop diverticulitis within their lifetime^4,5^. DD is the most common gastrointestinal cause for emergency department visits and inpatient admission, creating a significant health economic burden^6^. Over the past decade an increase in the number of elective operations for diverticulitis reflects the rising incidence of DD in a younger patient cohort^7^. Across Europe, DD accounts for around 13 000 deaths per year^8^.

The current understanding of the genomic predisposition for diverticulitis is founded on epidemiological studies. These studies identified several risk factors including: age, sex, obesity, sedentary lifestyle, dietary factors, alcohol, smoking, medication, co-morbidities and location^6,9–15^. However, environmental factors have not been able to risk stratify patients for management or reduce the disease burden. Further studies are being undertaken to better understand the pathophysiology of DD and its molecular pathways to evaluate the strength of associated risk factors.

Approximately 50% of the cause of DD is due to genetic effects, such as Mendelian connective tissue disorders like Ehlers–Danlos syndrome, polycystic kidney disease, Marfan’s syndrome and Williams–Beuren syndrome^16–18^. The aetiological trigger for symptomatic diverticulitis in diverticulosis patients remains unknown. Understanding the risk factors, pathophysiology and genetic predisposition to diverticulitis are important to direct clinical management in symptomatic patients^19,20^. There is variation in the management of DD including traditional dietary recommendations, antibiotics, and elective colectomy, alternative surgical approaches having fallen in and out of vogue in the last decade^21^. Approximately 15% of patients with diverticulitis require surgery and these operations are predominantly performed under emergency conditions that may result in the formation of a colostomy. Emergency operations for diverticulitis have high morbidity and mortality rates (reported up to 30%), a significant impact on quality of life and potential reoperation for stoma reversal in appropriate patients^22^.

The increasing incidence of diverticulitis, attributed to westernized industrialization compounding individual and environmental risk factors, places a strain on healthcare systems that makes understanding the disease and identifying patients that will progress to complicated disease increasingly important^8^. Understanding the causative genomic and molecular pathways creates the potential to identify a biological profile to predict complicated diverticulitis, which requires higher acuity of care or preventative treatment strategies such as surgery. This systematic literature review details the known genomic and epigenetic factors related to the formation of diverticulosis, highlighting the pathways indicated in association with diverticulitis.

## Methods

### Protocol and registration

The title, methods and outcome measures were stipulated in advance and can be found in the *Supplementary materials* (*Supp. S1*). There was insufficient evidence to perform a diagnostic accuracy, methodological or prognostic systematic review, therefore, this study was not registered on PROSPERO (an international prospective register of systematic reviews). A systematic review was performed to map evidence of the topic, identify main concepts and knowledge gaps, and examine the evidence in question to aid the planning and commissioning of future research in accordance with the PRISMA-ScR checklist (*Supplementary materials*, *Supp. S2*)^23^.

### Included studies

All papers including scientific evidence of colonic DD, epigenetics and human genetics were included with a focus on the genomics of inflammation and sepsis in diverticulitis. No regional restriction was placed. All study designs were considered for inclusion. Full-text papers available in the English language were included. Studies from 1972 up until the last search on 13 June 2023 were included.

### Participants

All studies reporting patients with diverticulosis, DD or diverticulitis were included. Studies pertaining to ‘segmental colitis associated with diverticulosis’ (SCAD) were excluded. All degrees of disease severity were included. No age restriction was applied but the studies included mainly focused on adult participants. Animal studies were excluded.

### Variables of interest

Genomics already known to be associated with DD in the literature were included, for example *ARHGAP15, FAM155A, COLQ*, and *TNFSF15* as well as epigenetic or genetic changes associated with DD. The correlation between genetic/epigenetic changes and the presence or severity of DD was assessed.

### Search strategy

The MEDLINE, PubMed, Embase, CINAHL (Cumulative Index to Nursing and Allied Health Literature) and Cochrane databases available through the National Health Service (NHS) National Library of Health website, the Cochrane library and PubMed available online were used. No time limit was placed on publication date. The last search was performed on 13 June 2023.

All titles containing the text and MeSH (Medical Subject Headings) search terms: ‘diverticular disease,’ ‘diverticulosis,’ ‘diverticulitis,’ ‘genomics,’ ‘genetics’ and ‘epigenetics’ were screened. Relevant papers identified in the database were included. Grey literature was searched using Google Scholar, personal communication, conference abstracts and abstract data. Full texts were reviewed, and quality was assessed by two independent reviewers. Studies relating to extra colonic diverticula were excluded. All relevant titles were then further screened by abstract. The full articles of eligible abstracts were then obtained. Bibliographies of the identified articles were manually screened for additional papers of interest. A PRISMA-ScR chart of the literature is shown (*Supplementary materials, Supp. S2*).

### Study selection and data collection process

Each included article was reviewed by three researchers. For abstracts with ambiguous relevance, full texts were screened. Where more specific or missing data was required the authors of manuscripts were contacted. Any disagreements that arose between the reviewers were resolved through discussion and if no consensus could be reached the senior author would decide. Data was entered onto an Excel worksheet.

### Data items and extraction

Appropriate scientific evidence on the genetics and epigenetics of DD was extracted. Patient demographics and study characteristics were extracted from the relevant studies. Evidence of good quality has been included for discussion in this article. Risk of bias and quality was discussed but was not able to be formally assessed due to the lack of studies available and inappropriateness of performing a quality assessment on too few studies.

## Results

**Fig. 1:**
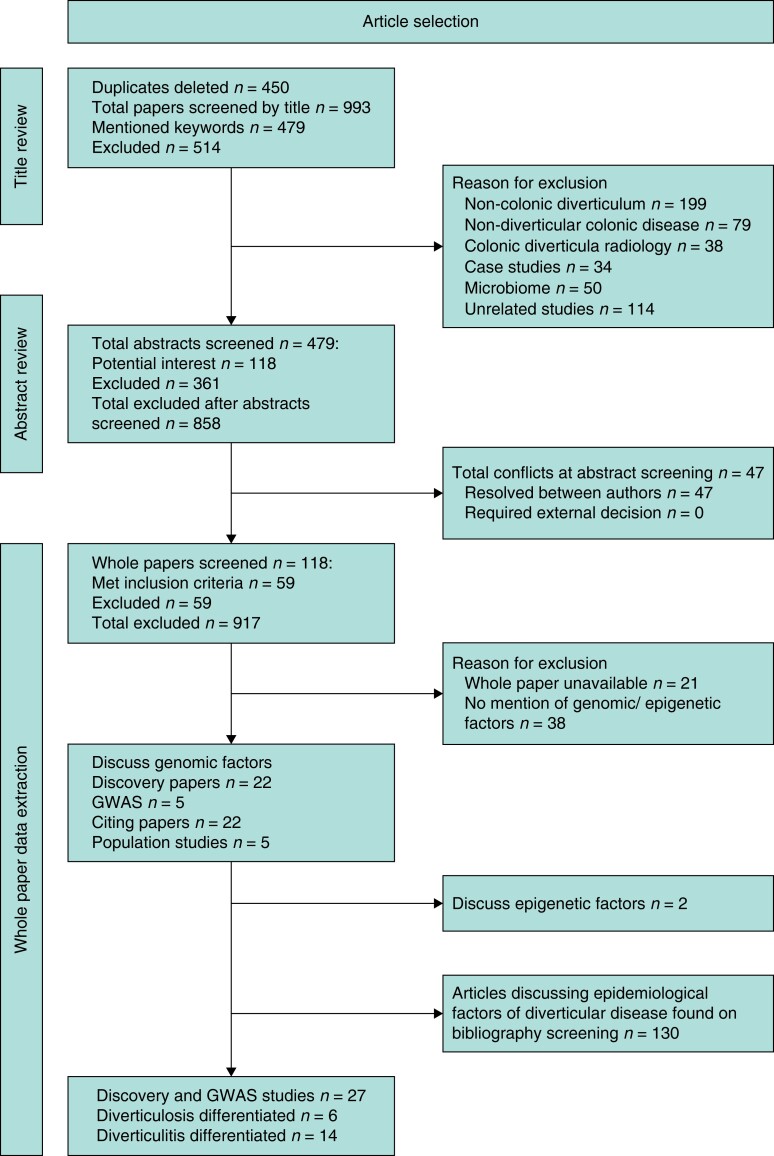
**PRISMA flow diagram** GWAS, genome-wide association studies.

The search strategy identified 995 articles, excluding duplicates. After screening of titles and abstracts, 59 articles met the inclusion criteria for the study. A further 130 articles of interest were found on manual screening of the bibliographies relating to epidemiology of DD (*Fig. 1*). A total of 27 articles reported discovery studies of potential novel mutations associated with DD (*Supplementary materials*, *Supp. S3*).

The literature review included five genome-wide association studies (GWAS), five population studies, 22 discovery studies and 22 citing papers. The level of evidence for each study, location and study population demographics are described (*Supplementary materials*, *Supp. S4*, *S5*, *S6*)^4^.

**Fig. 2:**
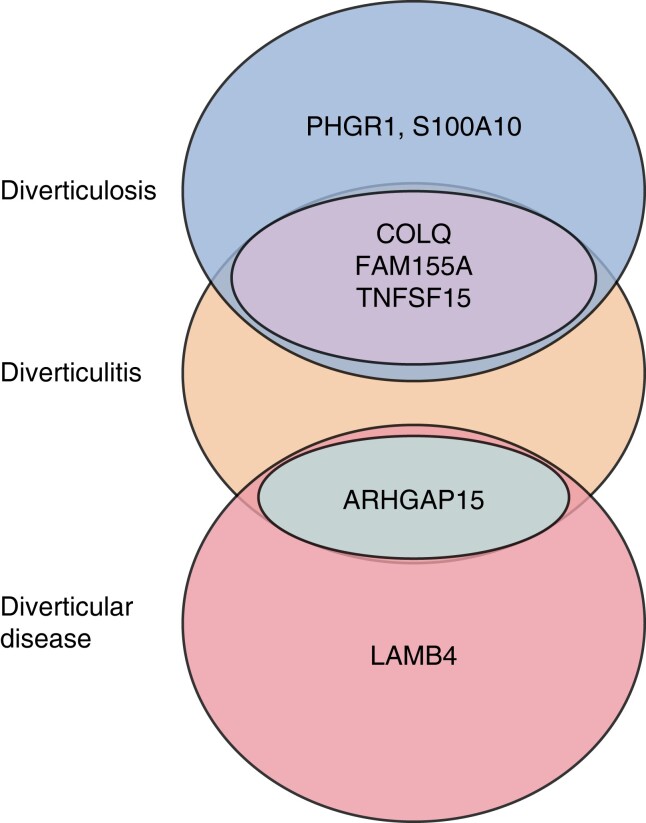
Schematic overlap of genes with strongest association to diverticulosis, diverticulitis and diverticular disease

A total of 86 novel loci have been described, of which 31 have been replicated. Mutations in 18 loci have been associated with diverticulosis, and 25 related to diverticulitis specifically (*Supplementary materials*, *Supp. S3*). The most cited loci were *ARHGAP15, COLQ, FAM155A*, and *TNFSF15*. The loci with the strongest association with DD are *ARHGAP15* and *LAMB4.* The loci with the strongest association with diverticulitis are *COLQ, FAM155A, PHGR1, S100A10*, and *TNFSF15* (*Fig. 2*).

Molecular pathways involved in extracellular matrix, connective tissue formation, immunity, membrane transport, cell adhesion and intestinal motility have been proposed in the literature as potential causative physiology in the development of diverticulosis. Some mutations are associated with pathways within the glial cell line-derived neurotrophic factor (GDNF) system, wingless-related integration site (WNT) signalling pathway, OAS 1/3 (2' - 5' oligoadenylate synthetase) pathway and RHOU pathway, downstream phenotypic applications described (*Supplementary materials*, *Supp. S6*).

Two papers identified in this study, from the same authors, described epigenetic changes associated with DD^24,25^.

## Epidemiology

### Age and sex

DD has traditionally been considered a disease of older adults with a peak incidence over the age of 70. There has been a large increase in prevalence in the 18–40 age group in a European cohort noted since 2000, in which the incidence per 1000 population increased from 0.15 to 0.251 in only 7 years, with no change being described over this time interval for those over 65^7,8,10,26^. In those over 50, DD has an equal prevalence between sexes. In the under 50 cohort, DD has a male predominance, with young males more likely to have aggressive disease, complications and higher risk of recurrence^11,12^. It is hypothesized that the prolonged time course of the bowel wall to insult, rather than age *per se*, increases the risk of diverticulosis. Caucasian Western patients have equal distribution between the sexes^27,28^. A Korean study showed an incidence by sex ratio of 2.2:1 male:female^29^.

It is reported that individuals with a family history of diverticulitis have a similar risk of recurrence when compared with those without a family history (HR 1.0; 95% c.i. 0.8 to 1.2) but are more likely to undergo elective surgery (HR 1.4; 95% c.i. 1.1 to 1.6). This was most notable in those with a first-degree family member affected by diverticulitis (HR 1.7; 95% c.i. 1.4 to 2.2)^30^.

### Obesity and sedentary lifestyle

The relationship between body mass index (BMI) and diverticulosis has conflicting evidence^13,14^. A large prospective series linked an increased BMI to an elevated risk of diverticulitis^31^. However, other population studies have not replicated this and found no association between BMI and diverticulitis at colonoscopy^4,32^. Physical activity has a significant correlation with reducing complications of diverticulitis^15–17^.

### Dietary factors

Studies attributing diverticulitis to low fibre, seeds and nuts have been disproved and no association has been supported^17,18^. The Professionals Follow-up Study (HPFS) comprising 51 000 US male health professionals found an inverse association between nut and popcorn consumption and the risk of diverticulitis with a reduction in the risk of diverticulitis and diverticular bleeding^33^.

The recommended dietary fibre intake for adults is 20–35 g/day, which few achieve by those consuming a ‘westernized’ diet. The Vitamin D and Calcium Polyp Prevention Study did not find an association between fibre and diverticulosis at colonoscopy^34,35^. However, secondary analysis using the Diet and Health Study and the Healthcare Professional Follow Up Study found patients with the highest fibre intake were most likely to have symptomatic diverticular disease^8,21,26^. Conversely, the Oxford cohort of the European Prospective Investigation into Cancer and Nutrition (EPIC) consisting of 57 000 patients from the UK found a protective effect of a fibre-rich diet^36^. This finding is reinforced by data from the prospective Million Women Study from the UK, which observed a reduced incidence of DD on a high fibre diet^37^. However, this depends on the specific sources of fibre, with the lowest risk resulting from fruit consumption^38^. Vitamin D regulates intestinal proliferation and is important to maintain colonic homeostasis by modulating inflammation. A study demonstrated that low ultraviolet (UV) light exposure is associated with diverticulitis and higher levels of 25-hydroxyvitamin D are associated with a lower risk of diverticulitis^39,40^.

There is conflicting evidence around the association with red meat, alcohol and smoking^14,19^. The Health Professionals Follow-Up Study found that red meat consumption in a westernized diet was associated with increased risk of incident diverticulitis and the EPIC-Oxford Study found red meat eaters had a higher risk of hospitalization from diverticulitis than vegetarians^36,41,42^. The EPIC-Oxford study, a prospective cohort of 47 678 US men, 40–75 years old, also found that those who drink > 30 g of alcohol/day have a weak and non-significant association with symptomatic DD when compared with non-drinkers. It is acknowledged that due to the multifactorial and slow course of the disease it is hard to study lifestyle factors over the interval in which diverticula develop.

### Co-morbidities

Hypertension and type 2 diabetes mellitus have been associated with asymptomatic diverticulosis^20,21,43^. The use of non-steroidal anti-inflammatory drugs (NSAIDs) and aspirin is associated with diverticular bleeds^22^. Steroids and opiate use are suggested to increase the presence of diverticula^1,44,45^. Smoking has not been significantly associated with the presence of diverticula^44,46,47^. Several case reports linking familial syndromes to increased severity of DD support the hypothesis that collagen tissue abnormality plays a role in the pathophysiology of diverticulosis (*Table 2*). However, studies investigating ancestry present conflicting data about the increased prevalence of DD in individuals with a positive family history. Some studies do show that individuals with a strong family history are more likely to have surgical management for DD^30,55,56^ (see *Table 1* for extrinsic factors related to DD).

**Table 1:** Extrinsic factors for diverticular disease

Risk factor	Potential risk factor	Potential protective factor	No effect	Potential risk factor
Age	Hypertension	Vegetarianism	Sex	Family history
Obesity	Type 2 diabetes mellitus	Exercise		Red meat consumption
Smoking	Medication: non-steroidal anti-inflammatory (including aspirin), steroids	Nuts, corn, seeds		Fibre
		High levels of vitamin D		Alcohol
		Medication: statins, probiotics		Microbiome profile

**Table 2.** Familial syndromes associated with increased risk of diverticulosis

Ehlers–Danlos syndrome (EDS) type IV^48,49^
Williams–Beuren syndrome^50^
Autosomal dominant polycystic kidney disease (ADPKD)^51^
Coffin–Lowry syndrome^52^
Marfan syndrome^53,54^

### Location

DD is found throughout the world and has become increasingly common notwithstanding global variation. South-East Asia reports predominantly right-sided true diverticulosis of 70–98% of all cases, peaking in the fifth decade with an overall prevalence between 8 and 25%^10^. Western populations have predominantly left-sided ‘false’ diverticulosis^11,12^ with over half of DD confined to the sigmoid colon^13,14^. False diverticula are an ‘out pouching’ of the mucosa/submucosa as compared with ‘true’ diverticula that contain all layers of the bowel wall.

**Fig. 3:**
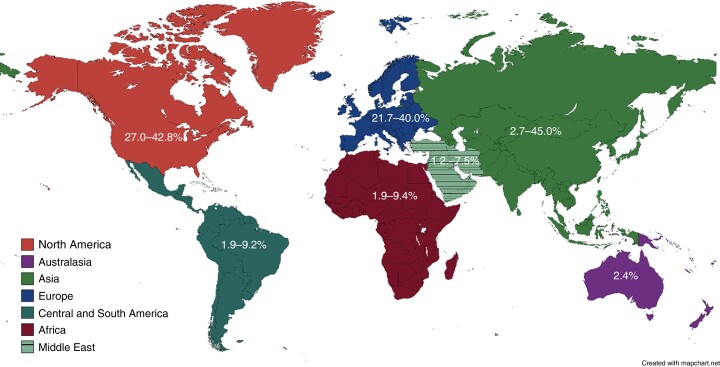
Percentage prevalence of the presence of diverticulosis at colonoscopy cited in the literature by location

North America has the highest incidence of DD worldwide, reaching 50% in the population over 60 years of age, followed by western Europe and Australia^57^. In contrast, Nigeria, Kenya and Egypt report the incidence of diverticulosis at colonoscopy as low as 9.4%, 6.6% and 2% respectively, although this may be biased by overall life expectancy^15,58,59^. Reports from Asia report a prevalence between 8 and 28.5%, which is variable depending on location and ethnicity, with a peak in the fifth decade of life^60–63^. Studies undertaken in Asia found DD affects the right colon in 70–98% of cases^60^. However, in Japan there has been a notable increase in prevalence (*P* < 0.01) from 66.0% in 2003 to 70.1% in 2011 in those aged over 60 and a higher prevalence of left-sided diverticulosis that is associated with the adoption of a westernized lifestyle^46^, *Fig. 3*.

In Europe, the prevalence of diverticulosis among white Western patients is 15–35% at colonoscopy, more frequently left-sided in older patients^13,64–66^. However, a lower incidence has been shown in developing countries^67^. There is no large population data from the Arabic or South American countries.

### Intrinsic factors

Disordered collagen and elastin are associated with diverticulosis. Increased rigidity and loss of tensile strength of the bowel wall is associated with densely packed collagen fibres with an overexpression of collagen cross-linking. Elastosis of the longitudinal muscle layer causes subsequent bowel wall thickening^68^.

Neuromuscular activity is affected by reduction in the myenteric plexus neurons and decreased myenteric glial cells and interstitial cells of Cajal with denervation hypersensitivity noted; this can cause uncoordinated contractions, high colonic pressure and associated muscle atrophy^26,28^. Neuromuscular colonic dysregulation may trigger symptoms of abdominal pain and cramping often associated with DD. Patients have a higher prevalence of visceral hypersensitivity^45,69^. Colonic motility is affected by serotonin levels and a significant decrease in 5-hydroxytryptamine (5HT) transporter (SERT) transcript levels has been found in the mucosa of patients with diverticulitis^11,29^.

## Genetics

### Extracellular matrix

The extracellular matrix (ECM), containing collagen, elastin and proteoglycans, maintains the integrity and flexibility of the colonic wall. Association with other connective tissue disorders such as rectal prolapse, polycystic kidney disease, heritable syndromes (Marfan or Ehlers–Danlos), female genital prolapse, aneurysm, inguinal hernia, hiatus hernia and joint dislocations points to an inherent underlying weakness in the connective tissue^70,71^. To maintain structure collagen forms cross-links; however, excessive cross-linking causes rigidity and loss of tensile strength, which has been associated with a low fibre diet with specific alterations in collagen demonstrated in diverticulosis^72^.

Ehlers–Danlos syndrome is a group of 13 inherited connective tissue disorders arising through mutations in *COL5A1* or *COL5A2* genes that partially encode type V collagen protein or the ECM protein tenascin-X^73^. Similarly, single nucleotide polymorphisms (SNPs) in *COL3A1*, with alteration in the collagen vascular system associated with inflammation, have been most strongly associated in Caucasian men when diverticulosis is present^19,74,75^. SNP missense variations in *COLQ* were found in both the Icelandic and Danish population studies; although the association is not of genome-wide significance, the replicable probability established by the association of rs7609897-T and the suggested association of rs146687198-G points to *COLQ* as a causative gene^76^. *COLQ* mutations can cause muscle weakness by reducing available acetylcholinesterase (AChE) at the neuromuscular junction; *COLQ* encodes a subunit of a collagen-like molecule that anchors AChE to the basal lamina. In addition, mutations in the *ELN* gene, encoding elastin, have been found in association with diverticulosis^77^.

Matrix metalloproteinases (MMP) are a family of homologous zinc-dependent endopeptidases. MMP 1, 8, 13 and 18 form the subgroups of collagenases. Activation of MMP results in an enzymatic cascade resulting in degradation and remodelling of the ECM including collagen, proteoglycans and other glycoproteins. Dysregulation of MMP has been shown to affect gut healing in several colonic diseases, for example enteric fistula formation in inflammatory bowel disease (BD)^74,78^.

Whilst a collagen deficit does not identify individuals who will go on to develop diverticulitis, one study demonstrated downregulation of MMP2, MMP9 and MMP13 expression and an increase in MMP1, TIMP1 and TIMP3 in inflamed mucosa of patients with diverticulitis *versus* Crohn’s disease. TIMP1 and TIMP3 are tissue inhibitors of metalloproteinases that affect the turnover of the ECM by controlling MMP activity^79,80^. The pathophysiological correspondence between diverticulitis and IBD is still unclear, with clinical overlap most notable in segmental colitis associated with diverticulosis (SCAD)^81^. Whilst the two diseases appear genetically separate, they share pathways of inflammation and fibrosis. At a molecular level, patients with IBD and diverticulitis were found to have similar levels of the inflammatory protein syndecan-1 (SD1) but significantly higher expression of basic fibroblastic growth factor (bFGF) and tumour necrosis factor (TNF-α) in diverticulitis^82–84^.

Furthermore, *LAMN4*, the laminin β-4 gene, localizes to the myenteric plexus of colonic tissue, a constituent of the ECM that has a role in regulation, development and colonic dysmotility. Two variants in this gene caused a reduction in laminin in a cohort of diverticulitis compared with controls^77,85^.

Growth factor genes *EGF, IGFI, CSF1, PDGF*, and *TPH-1* were found to have no significant association with DD^86,87^ (see *Table 2* for familial syndromes associated with diverticulosis).

### Neuromuscular function and motility

Neuromuscular function is intrinsically linked to the signalling pathways and downstream effects of the ECM. Histological staining has shown an increase in collagen fibres between the smooth muscle bundles in diverticulosis, associated with a decrease in *TACR1* and *TACR2*, which encode the NK2 receptor^88^.

Compared with controls, thicker circular and longitudinal muscle layers were present in DD with an increased connective tissue index and smooth muscle alterations^89^. This has been linked to variation in mRNA expression; deficiency in the GDNF system results in decreased SNAP-25 (synaptosomal-associated protein) and GDNF mRNA expression producing diminished immunoreactive signals in the enteric ganglia and a significant reduction in GFRa1 and RET (rearranged in transfection) receptors which code tyrosine kinase respectively^90–92^. The mRNA of Phox2b, expressed in the myenteric neuronal and glial part of the enteric nervous system, was increased in patients with diverticulitis^92^.

The prime gene candidates of interest among gene variants associated with alteration in neuromuscular function are *ANO1, PPP1R14A, COLQ6, COL6A1, CALCB* and *CALCA*, of which *ANO1* affects muscle contraction by influencing calcium-activated chloride channels in the pacemaker interstitial cells of Cajal^93^. *COL6A1*, along with *ARHGAP15* and *S100A10*, are proposed to impact the structure and tensile strength of smooth muscle; however, the SNPs and SNVs (single nucleotide variants) identified thus far in relation to these genes are mostly intronic and of unclear clinical significance^94^.

Several motility studies have shown dysregulation in association with DD. For example, upregulation of the nitrergic pathway has been recorded in early stages of diverticulosis and may play a role in colonic motor disorders^95^. Similarly, serotonin is a primary trigger for peristalsis, secretion and visceral sensation, dysregulation of which is observed in IBD and irritable bowel syndrome (IBS) in addition to diverticulitis. *Smoothelin-A* is a marker protein for smooth muscle contraction, inactivation of which results in a decrease in contractile potential, slow irregular wave patterns and colonic obstruction in knockout of the *Smoothelin* gene in a mouse model^96^.

### Vascular alteration

The genes *ELN, BMPR1B*, and *EFEMP1* are associated with connective tissue laxity, which may predispose to shearing of the culprit artery in diverticular bleeds. The *ABO* gene, related to the blood group system, encodes carbohydrates expressed on the surface of many tissues, increasing the risk of thrombosis and haemorrhage, in particular gastrointestinal bleeding. Similarly, *P2RY12* has a role in platelet function. Other genes associated with vascular disease include *CALCB, SLC35F3* and *CACNB2*, supporting the hypothesis that vascular disease drives diverticulitis^94^. *CALCB* is also associated with afferent nerve function and calcium balance, suggesting a role for dysmotility in diverticulitis pathophysiology.

The *WNT* signalling pathway, regulating cellular morphology and proliferation, is another key influence associated with DD, mediated by the *RHOU* gene that also has proangiogenic and human endothelial progenitor functions. *WNT4* is related to vascular smooth cell proliferation and this potentially supports the link between smooth muscle hypertrophy and atherogenesis in diverticulosis. The WNT family proteins are also reported to differentiate intestinal neuronal and glial cells and play a role in anti-inflammatory activity in a rat model, suggesting that *WNT* family genes play a pivotal role in the development of diverticulosis and the potential complication of diverticular bleeding^97^.

The clinical implications of genetic effects on the vasculature are evident in the impact of Nicorandil. First prescribed in 1984 for angina, reports of gastrointestinal ulceration and fistulation led to restriction of the drug. Data has shown that individuals with DD are at an increased risk of bowel perforation and seven times more likely to develop a fistula. It has been hypothesized that toxic Nicorandil metabolites accumulate in the blood vessels that often occur as the focal point of weakness within a diverticulum and cause ulceration, or the nitrous oxide released by the proinflammatory Nicorandil may trigger fistula formation among sigmoid diverticula. It has been proposed that genotyping may be used to guide prescribing by stratifying the individual risk of side effects^96,98^.

### Immunity

Inflammatory pathways have undergone less scrutiny thus far. Hub genes *RASAL3, SASH3, PTPRC*, and *INPP5D* were found to upregulate immune response-associated transcripts in the sigmoid colons of chronic, recurrent diverticulitis patients as identified by Schiefer *et al*^25^. These interconnected hub genes affect a network of immune regulators. *RASAL3* regulates natural killer T cell expansion and function in mouse models. *INPP5D (SHIP1)* is a leucocyte-specific molecule which negatively regulates phosphatidylinositol-3 kinase; it is proposed that upregulation of dephosphorylation affects signalling in the AKT (serine/threonine-protein kinase) pathway, increasing the rate of apoptosis. Reduced levels were found in intestinal biopsies of patients with Crohn’s disease and increased levels have been demonstrated in diverticulitis. *PTPRC (CD45)* encodes a cell-surface glycoprotein involved in protein tyrosine phosphorylation, in particular the initiation of leucocyte-specific immune responses resulting in high levels of inflammatory cytokines which may play a role in the chronicity of diverticulitis.

GWAS on the Icelandic, Denmark and UK populations replicated gene variants in *ARHGAP15, COLQ* and *FAM155A* with increased association with diverticulitis over diverticulosis^76^. Variants in *ARHGAP15*, the gene for Rho GTPase (guanosine triphosphate) activating protein 15, are associated with phagocyte function and inflammation; the role of *FAM155A* is unclear but is proposed to have a protective role against infection and inflammation^6^. The relative genetic risk of 27 replicating loci in a European cohort identified that *PHGR1, FAM155A-2, CALCB* and *S100A10* had a stronger effect in patients with diverticulitis as opposed to diverticulosis. Variants in *PHGR1* may induce inflammation by causing epithelial dysfunction and increasing the likelihood of bacterial penetration. *S100A10* regulates the remodelling of the extracellular matrix, further supporting the proposal that dysmotility and colon wall weakness have a role in the pathogenesis of diverticulitis^94^.

Two smaller studies found an associated SNP in the *TNFSF15* gene in the subset of patients requiring surgery for diverticulitis; *TNFSF15* encodes a cytokine in the TNF family and has been associated with severe IBD^56,99,100^. Connelly *et al.* propose the SNP rs7848647 may have the potential as a marker for DD severity to assist surgical decision-making. A rare SNP encoding a D435N substitution in *LAMB4* has also been described in diverticulitis and is known to play a role in intestinal barrier function^74^. Rat models have identified SNP variants near the *OAS1/3* gene that are upregulated by cytotoxic insult and interferons; OAS encodes a family of proteins with antiviral and apoptotic effects, the induction of which can result in chronic low-grade inflammation in the colonic wall^97^.

The association between diverticulitis with an allergic histamine response was studied and showed strong expression of H1R and H2R mRNA found in the colonic epithelium of complicated diverticulitis compared with controls and non-complicated diverticulitis, characterized by increased histamine release and massive inflammatory infiltration. Histamine is known as a regulator of gastrointestinal functions, such as gastric acid production, intestinal motility and mucosal ion secretion^101,102^.

At the transcriptome level an enrichment of innate and adaptive immune system pathways is observed in the sigmoid tissue of patients with diverticulitis. Network analysis suggests that diverticulitis is associated with dysregulation of the immune system both genetically and histologically^92^. However, in-depth analysis of wound healing transcriptome factors showed no statistical correlation with diverticulitis, and several genes identified have an unknown molecular pathway, highlighting the need for further research^22,55^.

The interactions between the molecular pathways and the host microbiome have also been demonstrated to play an important role in gut health and the growing understanding in this area has changed guidelines around antibiotic usage for uncomplicated diverticulitis. However, the interaction of genomics with the microbiome is an exciting area of development^103^. For example, bacteria and fungi can induce interferon expression that may alter the expression of *OAS* genes. Patients with early-onset DD were found to have elevated expression of host genes involved in the antiviral response, implying that susceptibility to a viral pathogen may play a role in the development of diverticulitis^24^.

The potential role of microbiota composition in the pathogenesis of diverticulosis is under investigation. In a prospective study of 43 patients comparing the microbiota profiles of patients with asymptomatic diverticulosis *versus* controls who underwent colonoscopy, no difference was detected between the two groups^104^. This is supported by further observational studies in the literature that did not detect a significant difference between microbiota in asymptomatic diverticulosis (AD) and controls, suggesting that microbiota may not play any role in the development of colonic diverticula^5,105^. However, in one study the symptomatic uncomplicated diverticular disease (SUDD) group showed significant alterations of the microbiota profile, with a reduction in *Enterobacteriaceae* sp. and higher levels of *Bacteroides/Prevotella* sp. in the biopsies coinciding with a higher macrophage count. In the faecal analysis, SUDD patients exhibited a significant reduction of members of clostridium cluster IX, *Fusobacteriae* sp. and *Lactobacillaceae* sp.^106^. In another small case-control study 16 patients with DD showed significantly higher levels of *Enterobacteriaceae* sp. compared with 35 controls without any diverticula^107^. This suggests a potential role of microbiota in the development of symptoms, and the symbiotic and pathognomonic role of the gut microbiome requires further study^108^ (*Table 3* lists intrinsic factors related to DD).

**Table 3.** Intrinsic factors for diverticular disease

**Increased**
Gut motility
**Abnormal**
Collagen
Elastin
Enteric nervous system
**Decreased**
Serotonin

## Epigenetics

Epigenetics is an important area of research assessing the impact of environmental factors on gene expression such as methylation. There is little published evidence on the impact of epigenetics on diverticulosis or diverticulitis but there have been interesting studies demonstrating immune cell epigenetic-mediated susceptibility to sepsis^107^. Early epigenetic data suggest variation in gene expression within molecular pathways in early- and late-onset diverticulitis, with early-onset diverticulitis displaying an increased expression of antiviral response genes^25^. This built on earlier work, by the same team, which suggested the hub genes *RASAL3*, *SASH3*, *PTPRC* and *INPP5D* within the brown module eigengene were highly correlated (*r* = 0.67, *P* = 0.0004) with diverticulitis. This data was supported by downstream analysis showing that transcripts associated with the immune response were upregulated in adjacent tissue from the sigmoid colons of chronic, recurrent diverticulitis patients^25^.

Exploratory studies such as DAMASCUS (Diverticulitis management, a snapshot collaborative audit study)^109^, gut microbiome work and broader demographic profiles will further the understanding and impact of epigenetics in diverticulitis^110^.

## Discussion

The genomic understanding of the causation and pathophysiology of the spectrum of DD is an area of fervent research. There is evidence of the genomic risk associated with DD, approximately 50%, as demonstrated in the Twin Studies and, whilst the variety of literature is limited, the wealth of information provided by GWAS provides novel pathophysiological insight.

Four GWAS undertaken in the UK, Europe and Korea have identified 49 susceptible loci, 25 replicated, associated with DD^76,93,97,111^. Two discriminated between diverticulosis and diverticulitis. Mapping has identified significant loci expression in pathways of extracellular matrix, connective tissue formation, immunity, membrane transport, cell adhesion and intestinal motility. These associated loci are highly expressed in mesenchymal stem cells, connective tissue cells and vascular cells^77^. Four replicated loci, *ARHGAP15, COLQ, FAM155A* and *TNFSF15*, are highlighted to have a stronger association with diverticulitis and may have a role in inflammation.

Due to the heterogenous nature of the disease and confusion about the most appropriate management pathways, clear classification and nomenclature is paramount; this has often been ambiguous in the literature. Diverticulosis is defined as the anatomical presence of colonic diverticula and DD as clinically significant and symptomatic diverticulosis. The umbrella term diverticular disease covers symptomatic uncomplicated diverticular disease (SUDD), a subtype of DD characterized by persistent abdominal symptoms in the absence of macroscopic colitis or diverticulitis, and diverticular bleed. Diverticulitis is the presence of macroscopic inflammation within the diverticula and can be complicated or uncomplicated; uncomplicated diverticulitis is identified on CT scan showing signs of inflammation such as colonic wall thickness and mesenteric/pericolic fat stranding, and becomes complicated with the addition of abscess, peritonitis, obstruction, fistula or per rectal haemorrhage. The colonoscopic Diverticular Inflammation and Complication Assessment (DICA) and radiological Hinchey classifications for standardized diagnostic reporting are now widely used^6,7^. Another condition, SCAD, is associated with the spectrum of DD with a clinical overlap to IBD; however, it may be considered a clinically distinct entity with different pathogenesis and surgical management^8^.

The most substantial genetic information on DD has been provided by GWAS. Sigurdsson *et al*.^75^ performed GWAS on Icelandic and Danish populations; however, in the Danish population they did not have the clinical data to separate into diverticulosis, DD and diverticulitis subsets, whereas Schafmayer *et al*.^109^ used the International Classification of Disease 10 diagnostic coding. Using population cohorts may lead to higher inaccuracy in clinical data than a case-control cohort; however, the statistical power from increased cohort size often outweighs this bias. Participant age ranges were also not provided, which leads to the additional concern of misclassification in a potentially younger cohort who may yet develop a diagnosis of diverticulosis. Schafmayer *et al*.^109^ and Maguire *et al*.^91^ studied the UK Biobank, a prospective cohort study on ∼500 000 population-based individuals, with different hospital registers for comparison identifying 39 and 48 susceptibility loci respectively. GWAS loci usually harbour multiple genes, making the causal gene hard to identify. Candidate genes can be identified by downstream *in silico* analysis and additional analysis of mRNA expression and fluorescence immunohistochemical staining in colonic biopsies. However, due to the intronic nature of several gene variants identified, these mechanisms must be viewed hypothetically, in need of robust study to interrogate functional impact^102,112^.

There is a known ethnic variation between Eastern and Western populations and caution must be exercised interpreting the presence of mutations and generalizing data within the demographic context. Choe *et al*. performed a GWAS in Korea that identified three novel loci associated with *WNT, RHOU*, and *OAS 1/3*, which were not described in the Western cohorts of the other GWAS that has a greater overlap of identified loci. Large-scale epidemiological studies assessing the role of fibre were undertaken in the USA and Japan and had similar results^33,35^. These studies do not consider the socioeconomic background of the participants. Furthermore, studies reporting prevalence do not consider comparative life expectancy or access to healthcare and colonoscopy. This requires further exploration as the body of the literature has been undertaken in Western populations.

The multifactorial nature of DD presents difficulties in interpreting the specificity of complex genomic interactions and molecular pathways. Diverticulosis is associated with numerous co-morbidities including obesity, hypertension and age, which may increase mutation and affect phenotype. Awareness for the presence of simultaneous gastrointestinal diseases and other connective tissue disorders is also required; a candidate-gene study assessing colon cancer found an association between diverticulosis and a variant in *RPRM*, a tumour suppressor gene regulating the G2 arrest of the cell cycle^113^. Strikingly, there is minimal overlap between diverticulitis and other inflammatory bowel diseases^114^.

The crucial question yet to be determined is the distinction between causation and association for the identified, replicable loci. For example, serotonin is the primary trigger for gut motility, and it is unclear if the upregulation of *5HT-4R*, the gene associated with serotonin production, is a causative factor for diverticulitis or a phenotypic effect of localized inflammation^87^. Further studies are required to identify the causative genetic factor of diverticulosis and diverticulitis and associated genetic changes influenced by other risk factors. The ALADDIN (alpha-1-antitrypsin deficiency carriers in a population with and without colonic diverticula) study is underway to investigate A1AT deficiency, a protease inhibitor that protects connective tissue, as a causative risk factor for the development of diverticulosis^113^.

This review has a number of limitations, most notably the lack of meta-analysis due to the limited literature. It was not possible to analyse the replicability or strength of association for each mutation or demonstrate significant differences between different patient cohorts. Bias was not formally assessed in the papers, however, this is of less relevance, given the low level of evidence of the studies and citing articles excluding the higher quality GWAS. The limitations of this study highlight the need for robust, replicable research into DD that can be translated into clinical practice.

The evidence supports the importance of genomics as a crucial factor in the pathophysiology of diverticula formation and the risk of diverticulitis. *COLQ, FAM155A, PHGR1, ARHGAP15, S100A10*, and *TNFSF15* are the strongest candidates for further research to develop a tool to clinically stratify patients into surgical and conservative management. Including genomics there is a need for more ‘omic’ research in areas such as epigenomics, transcriptomics, microbiomics and metabolomics.

## Supplementary Material

zrae032_Supplementary_Data

## Data Availability

No novel data are included in this systematic review.
