# Supplementary material for: Genetic, epigenetic and environmental factors in diverticular disease: systematic review
Source: BJS Open. 2024 Jun 4;8(3):zrae032. doi: 10.1093/bjsopen/zrae032 (PMC11148476; doi:10.1093/bjsopen/zrae032)
Supplement: zrae032_Supplementary_Data [file zrae032_supplementary_data.docx]

**Epigenetic, genetic, and environmental factors in diverticular disease: a comprehensive review**

H N Humphrey^1^ (ORCID No. 0000-0002-1238-9741)

P Sibley^1^ (ORCID No. 0009-0005-4274-271X)

E T Walker^1^ (ORCID No. 0000-0003-2810-8200)

D S Keller^2^ (ORCID No. 0000-0002-8645-6206)

F Pata^3,4^ (ORCID No. 0000-0003-2634-1199)

D Vimalachandran ^5,6^ (ORCID No. 0000-0001-5817-8969)

I R Daniels^1^ (ORCID No. 0000-0002-9114-0812)

F D McDermott^1^ (ORCID No. 0000-0002-4891-1191)

^1^ General Surgery, Royal Devon University NHS Foundation Healthcare Trust, Barrack Road, Exeter, Devon, UK

^2^ Department of Colorectal Surgery, Lankenau Medical Center, Wynnewood, PA, USA

^3^ General Surgery Unit, Nicola Giannettasio Hospital, Corigliano-Rossano, Italy

^4^ Department of Pharmacy, Health and Nutritional Sciences, University of Calabria, 87036 Rende, Italy

^5^Institute of Cancer Medicine, University of Liverpool, Liverpool, UK

^6^ Department of Colorectal Surgery, Countess of Chester Hospital NHS Foundation Trust, Chester, UK

**Address for Correspondence:** Mr F McDermott MD FRCS Consultant Colorectal Surgeon, Royal Devon University Healthcare Trust, Barrack Road, EX2 5DW, Exeter, Devon, UK.

Email: [f.mcdermott@nhs.net](mailto:f.mcdermott@nhs.net)

**SUPPLEMENTARY MATERIAL**

| **Supplementary Figures and Tables** |  |
| --- | --- |
| **S1.** Search strategy.  **S2** PRISMA- ScR Table  **S3** Data characteristics of discovery studies identifying DNA/ mRNA mutations with potential associations to DD.  **S4** Overview of population studies reviewing the genetic susceptibility of a population to the presence of DD.  **S5** Papers citing DNA/ mRNA mutations associated with DD.  **S6** DNA/mRNA mutations associated with Diverticulitis/ Diverticulosis/ Diverticular Disease. | *Page 3*  *Page 7*  *Page 12*  *Page 21*  *Page 22*  *Page 24* |
| **References** | *Page 48* |

**S1.** Search strategy

| MEDLINE (OVID)  1. exp genetic testing/  2. exp genetics/  3. exp epigenetics/  4. exp DNA/  5. (epigenetic* or genetic* or genom* or genes or epigenom* or GWAS or methylom* or methylation or DNA or "deoxyribonucleic acid" or (genetic adj5 tissue) or (genetic adj5 "liquid biops*")).ti,ab.  6. 1 or 2 or 3 or 4 or 5  7. exp colon diverticulosis/ or exp diverticulosis/  8. exp diverticulitis/  9. (diverticulosis or diverticulitis or diverticular).ti,ab.  10. 7 or 8 or 9  11. 6 and 10  12. limit 11 to (medline and yr="1972 -Current") | |
| --- | --- |
| EMBASE (OVID)  1. exp genetic screening/  2. exp genetics/  3. exp epigenetics/  4. exp DNA/  5. (epigenetic* or genetic* or genom* or genes or epigenom* or GWAS or methylom* or methylation or DNA or "deoxyribonucleic acid" or (genetic adj5 tissue) or (genetic adj5 "liquid biops*")).ti,ab.  6. 1 or 2 or 3 or 4 or 5  7. exp colon diverticulosis/ or exp diverticulosis/  8. exp diverticulitis/  9. (diverticulosis or diverticulitis or diverticular).ti,ab.  10. 7 or 8 or 9  11. 6 and 10  12. limit 11 to (embase and yr="1972 -Current") | |
| PubMED  1. exp genetic testing/  2. exp genetics/  3. exp epigenetics/  4. exp DNA/  5. (epigenetic* or genetic* or genom* or genes or epigenom* or GWAS or methylom* or methylation or DNA or "deoxyribonucleic acid" or (genetic adj5 tissue) or (genetic adj5 "liquid biops*")).ti,ab.  6. 1 or 2 or 3 or 4 or 5  7. exp colon diverticulosis/ or exp diverticulosis/  8. exp diverticulitis/  9. (diverticulosis or diverticulitis or diverticular).ti,ab.  10. 7 or 8 or 9  11. 6 and 10  12. limit 11 to (yr="1972 -Current") | |
| CINAHL  1. exp genetic screening/  2. exp genetics/  3. exp epigenetics/  4. exp DNA/  5. (epigenetic* or genetic* or genom* or genes or epigenom* or GWAS or methylom* or methylation or DNA or "deoxyribonucleic acid" or (genetic adj5 tissue) or (genetic adj5 "liquid biops*")).ti,ab.  6. 1 or 2 or 3 or 4 or 5  7. exp diverticulum colon/ or exp diverticulosis/  8. exp diverticulitis/  9. (diverticulosis or diverticulitis or diverticular).ti,ab.  10. 7 or 8 or 9  11. 6 and 10  12. S6 and S11 | |
| COCHRANE  1.MeSH descriptor: [Genetic Testing] explode all trees  2.MeSH descriptor: [Genetics] explode all trees  3.MeSH descriptor: [Epigenomics] explode all trees  4.MeSH descriptor: [DNA] explode all trees  5.(epigenetic* or genetic* or genom* or genes or epigenom* or GWAS or methylom* or methylation or DNA or "deoxyribonucleic acid" ):ti,ab  6.#1 or #2 or #3 or #4 or #5  7.MeSH descriptor: [Diverticulosis, Colonic] explode all trees 108  8.MeSH descriptor: [Diverticular Diseases] explode all trees  9.MeSH descriptor: [Diverticulitis, Colonic] explode all trees  10.MeSH descriptor: [Diverticulitis] explode all trees  11.#7 ot #8 or #9 or #10  12.#6 and #11 | |
| Eligibility Criteria | |
| Inclusion criteria:   1. Full text available in English language 2. Includes human genetic, genomic and/ or epigenetic factors of colonic diverticular disease 3. Adult and animal studies with reference to human models | Exclusion Criteria:   1. Full text unavailable in English Language 2. Case studies and abstracts 3. Non- colonic diverticulum 4. Animal studies unrelated to human models 5. Microbial DNA/ RNA |
| Data charting process and outcome measures | |
| 1. Trends of extrinsic factors for Diverticular Disease (DD). 2. Trends of Intrinsic Factors for Diverticular Disease. 3. Familial syndromes associated with presence of diverticulosis. 4. Data characteristics of discovery studies identifying DNA/ mRNA mutations with potential associations to DD including, authors, PMID, year, location, study population size, age and gender demographics as cited in study papers identified. (S2) 5. Overview of population studies reviewing the genetic susceptibility of a population to the presence of DD. (S3) 6. Papers citing DNA/ mRNA mutations associated with DD. (S4) 7. DNA/mRNA mutations associated with Diverticulitis/ Diverticulosis/ Diverticular Disease. (S5) | |

**S2** Preferred Reporting Items for Systematic reviews and Meta-Analyses extension for Scoping Reviews (PRISMA-ScR) Checklist

| **SECTION** | **ITEM** | **PRISMA-ScR CHECKLIST ITEM** | **REPORTED ON PAGE #** |
| --- | --- | --- | --- |
| **TITLE** | | | |
| Title | 1 | Identify the report as a scoping review. | 1 |
| **ABSTRACT** | | | |
| Structured summary | 2 | Provide a structured summary that includes (as applicable): background, objectives, eligibility criteria, sources of evidence, charting methods, results, and conclusions that relate to the review questions and objectives. | 3 |
| **INTRODUCTION** | | | |
| Rationale | 3 | Describe the rationale for the review in the context of what is already known. Explain why the review questions/objectives lend themselves to a scoping review approach. | 4-5 |
| Objectives | 4 | Provide an explicit statement of the questions and objectives being addressed with reference to their key elements (e.g., population or participants, concepts, and context) or other relevant key elements used to conceptualize the review questions and/or objectives. | 5 |
| **METHODS** | | | |
| Protocol and registration | 5 | Indicate whether a review protocol exists; state if and where it can be accessed (e.g., a Web address); and if available, provide registration information, including the registration number. | 6 |
| Eligibility criteria | 6 | Specify characteristics of the sources of evidence used as eligibility criteria (e.g., years considered, language, and publication status), and provide a rationale. | 6 |
| Information sources* | 7 | Describe all information sources in the search (e.g., databases with dates of coverage and contact with authors to identify additional sources), as well as the date the most recent search was executed. | 6-7 |
| Search | 8 | Present the full electronic search strategy for at least 1 database, including any limits used, such that it could be repeated. | Supplementary Material. Figure 1. S1 |
| Selection of sources of evidence† | 9 | State the process for selecting sources of evidence (i.e., screening and eligibility) included in the scoping review. | 6 |
| Data charting process‡ | 10 | Describe the methods of charting data from the included sources of evidence (e.g., calibrated forms or forms that have been tested by the team before their use, and whether data charting was done independently or in duplicate) and any processes for obtaining and confirming data from investigators. | 7 |
| Data items | 11 | List and define all variables for which data were sought and any assumptions and simplifications made. | 6-7, Supplementary Material. Figure 1. S1 |
| Critical appraisal of individual sources of evidence§ | 12 | If done, provide a rationale for conducting a critical appraisal of included sources of evidence; describe the methods used and how this information was used in any data synthesis (if appropriate). | N/A, formal, pre- structured critical analysis not undertaken, relevance and reliability of pertinent articles reviewed in discussion. |
| Synthesis of results | 13 | Describe the methods of handling and summarizing the data that were charted. | Authors HNH, ETW, PS, FDM agreed outcome data points, data collected and copied directly from source articles and supplementary material into Excel spreadsheets. Further statistical/ meta analysis not performed. |
| **RESULTS** | | | |
| Selection of sources of evidence | 14 | Give numbers of sources of evidence screened, assessed for eligibility, and included in the review, with reasons for exclusions at each stage, ideally using a flow diagram. | Supplementary Material. Figure 2. S9 |
| Characteristics of sources of evidence | 15 | For each source of evidence, present characteristics for which data were charted and provide the citations. | Supplementary Material. Table 4. S5 |
| Critical appraisal within sources of evidence | 16 | If done, present data on critical appraisal of included sources of evidence (see item 12). | N/A |
| Results of individual sources of evidence | 17 | For each included source of evidence, present the relevant data that were charted that relate to the review questions and objectives. | Supplementary Material. Table 5. S6, Table 6. S7, Table 7. S8 |
| Synthesis of results | 18 | Summarize and/or present the charting results as they relate to the review questions and objectives. | 7-18 |
| **DISCUSSION** | | | |
| Summary of evidence | 19 | Summarize the main results (including an overview of concepts, themes, and types of evidence available), link to the review questions and objectives, and consider the relevance to key groups. | 7-18 |
| Limitations | 20 | Discuss the limitations of the scoping review process. | 19-21 |
| Conclusions | 21 | Provide a general interpretation of the results with respect to the review questions and objectives, as well as potential implications and/or next steps. | 21 |
| **FUNDING** | | | |
| Funding | 22 | Describe sources of funding for the included sources of evidence, as well as sources of funding for the scoping review. Describe the role of the funders of the scoping review. | N/A |

JBI = Joanna Briggs Institute; PRISMA-ScR = Preferred Reporting Items for Systematic reviews and Meta-Analyses extension for Scoping Reviews.

* Where *sources of evidence* (see second footnote) are compiled from, such as bibliographic databases, social media platforms, and Web sites.

† A more inclusive/heterogeneous term used to account for the different types of evidence or data sources (e.g., quantitative and/or qualitative research, expert opinion, and policy documents) that may be eligible in a scoping review as opposed to only studies. This is not to be confused with *information sources* (see first footnote).

‡ The frameworks by Arksey and O’Malley (6) and Levac and colleagues (7) and the JBI guidance (4, 5) refer to the process of data extraction in a scoping review as data charting*.*

§ The process of systematically examining research evidence to assess its validity, results, and relevance before using it to inform a decision. This term is used for items 12 and 19 instead of "risk of bias" (which is more applicable to systematic reviews of interventions) to include and acknowledge the various sources of evidence that may be used in a scoping review (e.g., quantitative and/or qualitative research, expert opinion, and policy document).

*From:* Tricco AC, Lillie E, Zarin W, O'Brien KK, Colquhoun H, Levac D, et al. PRISMA Extension for Scoping Reviews (PRISMAScR): Checklist and Explanation. Ann Intern Med. 2018;169:467–473. [doi: 10.7326/M18-0850](http://annals.org/aim/fullarticle/2700389/prisma-extension-scoping-reviews-prisma-scr-checklist-explanation).

**S3** Data characteristics of discovery studies identifying DNA/ mRNA mutations with potential associations to DD.

| **First Author** | **PMID/ DOI** | **Year** | **Location** | **Level of Evidence** | **Total Popultation Size** | **Case Population** | **Controls Population** | **Male Cases** | **Male Controls** | **Age Cases (Years)** | **Age Controls (Years)** | **Sample** | **Disease Studied** | **Analyte** | **DNA Analysis** | **Mutation Gene Identified** |
| --- | --- | --- | --- | --- | --- | --- | --- | --- | --- | --- | --- | --- | --- | --- | --- | --- |
| **GWAS** |  |  |  |  |  |  |  |  |  |  |  |  |  |  |  |  |
| Joo (1) | 37196047 | 2023 | USA | I | 21,777 | 12,577 | 9200 | 45.40% | x | 62.5 | x | In vitro colonic tissue specimen | D-osis and D-itis | DNA | (RT-qPCR) GWAS and Phenome-wide association study (PWAS) | ARHGAP15 |
| Choe (2) | 31089239 | 2019 | Korea | I | 2619 | Test: 893 Replication 346 | Test: 1075 Replication: 305 | 65.50% | 72.90% | 61.5 | 54.6 (mean) | Blood | Right sided D-osis | DNA | (PCR) GWAS | *WNT4, RHOU, OAS1/3* |
| Schafmayer (3) | 30661054 | 2019 | UK, Germany, Austria, Germany, Sweden, UK | I | 457821 | Test: 31964 Replication: 3893 | Test: 419135 Replication: 2829 | 46.50% | 45.70% | 72 | 68 (mean) | In vitro colonic tissue specimen | D-osis and D-itis | mRNA and protein | (RT-qPCR) GWAS | 48 risk loci, of which 12 are novel (See Table X) |
| Maguire (4) | 30177863 | 2018 | Denmark, USA | I | 440949 | Test: 27444 ReplicationL 2572 | Test: 382284 Replication: 28649 | x | x | x |  | In vitro colonic tissue specimen | D-itis and Diverticular haemmorhage | mRNA | (RT-qPCR) GWAS and Phenome-wide association study (PWAS) | 42 loci associated with DD, 39 of them novel (See Table xx) |
| Sigurdsson (5) | 28585551 | 2017 | Denmark, Iceland, USA | I | 24210 | Test: DD 5426, D-itis 2764 Replication: 5970 | Test: 7030 Replication: 3,020 | 40% | 51% | 68 | 55.4 (mean) | Blood | DD and D-itis | mRNA | (RT-qPCR) GWAS | *ARHGAP15, COLQ and FAM155A* |
| **Cohort study** |  |  |  |  |  |  |  |  |  |  |  |  |  |  |  |  |
| Noyes (6) | 32535905 | 2020 | UK | II.1 | 18306 | 10149 | 8,157 | 57.00% | 61% | 68.8 | 67.7 (mean) | Blood | Risk of Nicorandil use in a T2DM population | DNA | Genotyping across 5 platforms: Affymetrix Genome-Wide Human SNP Array 6.0, Illumina HumanOmni Express, SHAPEIT14, IMPUTE2, and various custom genotyping arrays from Illumina | Not stated |
| Reichert (7, 8) | 29533249 | 2018 | Poland, Germany | II.1 | 707 | 422 | 285 | 49.50% | 49.10% | 68 (median) | 57 (median) | Blood | D-osis | DNA | Genotyping with Taqman assays and qPCR | *COL3A1, COL1A1* |
| Schieffer (8) | 30240468 | 2018 | USA | II.1 | 26 | 13 Early Onset <45 years (EO), 13 Late Onset >45 yeas (LO) | 52 | 50.00% | 60.1% (EO), 30.8% (LO) | 37.1 (EO mean), 73.2 (LO mean) | matched | Sigmoid colon tissue | D-itis | mRNA and epigenome | RT-qPCR and epigenetic hierarchical clustering analyisis weighted co-expression network analysis | *OASL, ISG15, IFIT1* |
| Schieffer (9) | 28619727 | 2017 | USA | II.1 | 25 | 20 | 5 | 50.00% | 40% | 57.3 | 57.1 (mean) | Sigmoid colon tissue | D-itis | mRNA and epigenome | RT-qPCR and epigenetic hierarchical clustering analyisis weighted co-expression network analysis | *RASAL3, SASH3, PTPRC, INPP5D, TBX21, BLKCCR7, TCL1A* |
| **Case- control study** |  |  |  |  |  |  |  |  |  |  |  |  |  |  |  |  |
| Reichert (10) | 32015353 | 2020 | Germany, Poland, Austria | II.1 | 1332 | 424 (d-osis), 105 (d-itis) | 844 | 49.4% (d-osis) 53.0% (d-itsis) | 44.3 (d-osis) 47.6 (d-itis) | 67 (d-osis) 61 (d-itis) (median) | 57 (d-osis) 64 (d-itis) (median) | Blood | D-osis and D-itis | DNA | Genotyping with Taqman assays | *ARHGAP15, COLQ, FAM155A* |
| Kline (11) | 30511196 | 2019 | USA | II.1 | 404 | 28 | 376 | 36% | 47% | 51 (mean) | 56 (mean) | Sigmoid colon tissue | D-itis | RNA | Gene set enrichment analysis |  |
| Connelly (12) | 28624054 | 2017 | USA | II.1 | 348 | 148 (90<50years 87>50years) | 200 | 49.30% | 29.50% | 59.1 (mean) | 51.9 (mean) | Blood | DD | DNA | Genotyping with Taqman assays | *TNFSF15* |
| Barrenschee (13) | 25655772 | 2015 | Germany | II.1 | 19 | 9 | 10 | 18% | 40% | 49–74 (range) | 48–90 (range) | Human colonic tissue and rat mucosa | DD | mRNA and protein | RT-qPCR | *SNAP- 25* |
| Dai (14) | 25666316 | 2015 | Australia | II.1 | 35 | 13 | 22 | 15% | x | 68 (median) | 50 (median) | Colonic tissue | D-itis | mRNA | qPCR and immunohistochemistry | *COX-1, COX-2, 15-PGDH* |
| Connelly (15) | https://dx.doi.org/10.1097  /SLA.0000000000000232 | 2014 | USA | II.1 | 94 | 63 Sporadic Diverticulitis (DD), 5 Familial Divertiuclitis (FD) | 26 | 54% (SD), 20% (FD) | 0% | 52 (SD), 51.1 (FD) (mean) | 57.7 (mean) | Blood | DD | DNA | Genotyping with Taqman assays | *TNFSF15* |
| Hellwig (16) | 24113817 | 2014 | Germany | II.1 | 39 | 20 | 19 | 40% | 53% | 40–74 (range) | 41–90 (range) | Colonic tissue | DD | mRNA and protein | RT-qPCR | *a-SMA, HDAC8, SM, SMMHC* |
| Bottner (17) | 23805210 | 2013 | Germany | II.1 | 41 | 20 | 21 | 55% | 33% | 60.8 (mean) | 71.3 (mean) | Colonic tissue | DD | mRNA | RT-qPCR | *GDNF, GFRα1, RET* |
| Rahden (18) | 21956435 | 2012 | Germany | II.1 | 85 | 41 | 44 | 53% | 45% | 56.9 (mean) | 61.5 (mean) | Colonic tissue | D-itis | mRNA | RT-qPCR | *H1R, H2R* |
| Liu (19) | 10.1111/j.1365-2982.2011.01685.x | 2011 | Australia | II.1 | 52 | 23 | 29 | 30% | x | 47–80 (range) 68 (median) | 21–81 (range) 58 (median) | Colonic tissue | Ulcerative colitis, Chron's Disease, DD | DNA and protein | PCR | *TACR1, TACR2* |
| Costedio (20) | 18491196 | 2008 | USA | II.1 | 51 | 13 D-osis, 19 D-itis | 22 | 56% D-osis, 30% D-it is | 45% | 59 (D-osis), 55 (D-itis) | 52 (median) | Colonic tissue | D-itis | mRNA | RT-qPCR | *SERT* |
| Rottier* (21) | 30767045 | 2019 | Netherlands | II.1 | 230 individuals (incomplete) | x | x | x | x | x | x | Blood | DD | DNA | Immunoassay and allele specific amplification Amplicon sequencing (Ampliseq) panel with coding sequences (CDS) | *SERPINA1, SERPINA3, ELA2* |
| **Case series and animal studies** |  |  |  |  |  |  |  |  |  |  |  |  |  |  |  |  |
| Niessen (22) | 16285958 | 2005 | Netherlands | II.2 | x | x | x | x | Mice | x | x | Colonic tissue |  | mRNA and protein | RT-qPCR | *SMTN* |
| Kline (23) | 34225052 | 2021 | USA | II.2 | 24 | x | x | x | x | x | x | Colonic tissue | DD and D-itis | DNA | qPCR and immunohistochemistry | *ARHGAP15, COLQ, FAM155A* |
| Cossais (24) | 31019703 | 2019 | Germany | II.2 | 40 | 20 | 20 | 35% | 65% | 63 (mean) | 69 (mean) | Sigmoid colon tissue | D-itis | mRNA | RT-qPCR | *PHOX2B* |
| Coble (25) | 28595269 | 2017 | USA | II.2 | 148 | x | x | 48% | x | 30 -83 (range) 58 (median) | x | Blood, saliva and colonic mucosa | D-osis | DNA | Exome sequencing | *LAMB4* |
| Ha Se (26) | 28806761 | 2017 | USA | II.2 | x | x | x | x | Mice | x | x | Colonic tissue | PDGFRα+ cell hyperplasia | mRNA and protein | RT-qPCR NGS | *CACNA1g* |
| Connelly (27) | 26003116 | 2015 | USA | II.2 | 46 | 27 | 19 | 45% | 100% | 39 (mean) | 52.9 (mean) | Sigmoid colon tissue | D-itis | mRNA | Qiagen™ Wound Healing Array | No association found |

**S4** Overview of population studies reviewing the genetic susceptibility of a population to the presence of DD.

| **First Author** | **PMID** | **Year** | **Location** | **Study** | **Level of Evidence** | **Total Sample Size** | **Susceptibility to DD Results from Genetic Factors** |
| --- | --- | --- | --- | --- | --- | --- | --- |
| Strate (28) | 23313967 | 2013 | USA | Population-based sample of twins | II.1 | 142,123 | 53% (95% CI, 45%–61%) |
| Granlund (29) | 22432696 | 2012 | Sweden | Population-based sample of twins | II.1 | 104,452 | 40% |
| Saylors (30) | https://doi.org/10.1007/s11605-023-05716-7 | 2023 | USA | Population- based cohort study | II.1 | 169,120 |  |
| Cohan (31) | 34933317 | 2021 | USA | Population-based cohort study | II.1 | 4426 |  |
| Broad (32) | 30719942 | 2019 | New Zealand | Population-based cohort study | II.1 | 3,564,727 |  |

**S5** Papers citing DNA/ mRNA mutations associated with DD.

| **First Author** | **PMID** | **Year** | **Location** | **Article** | **Level of Evidence** | **Citing Mutation** |
| --- | --- | --- | --- | --- | --- | --- |
| Mastoraki (33) | 33409567 | 2021 | Greece | Systematic Review | III | *GFRA1, RET, 5HT-4R, TACR2, TACR1, TNFSF15, LAMB4, RASAL3, SASH3, PTPRC, INPP5D, ARHGAP15, COLQ, FAM155A, COL3A1, CTAGE1, PHGR1, CALCB, and S100A10* |
| Peery (34) | 33279517 | 2021 | USA | Review | III | *PHGR1, FAM155A, CALCB and S100A10* |
| Maguire (35) | 32542557 | 2020 | USA | Review | III | *ARHGAP15, FAM155A, COLQ, ARHGAP15, ANO1, ELN, SPINT2, LAMB4 and TNFSF15* |
| Miulescu (36) | 32419870 | 2020 | Romania | Review | III | Not specific |
| Nasef (37) | 32635383 | 2020 | New Zealand | Review | III | *COL3A1c, ARHGAP15, COLQ, FAM155A, LAMB4, RASAL3, PTPRC, INPP5D, SAM andSASH3* |
| Tursi (38) | 32218442 | 2020 | Italy | Review | III | *COL3A1, TNFSF15, RPRM, LAMB4, ARHGAP15, COLQ, FAM155A* |
| Camilleri (39) | 31351939 | 2020 | USA | Review | III | *ABO, ANO1, CP-17 (aka PPP1R14A), COLQ6, COL6A1, CALCB or CALCA, COL6A1, ARHGAP15, PHGR1, FAM155A-2, ELN, P2RY12, SLC35F3, BMPR1B, EFEMP1 and S100A10, and LAMB4 gene* |
| Kupcinskas (40) | 31930230 | 2019 | USA, Lithuania, Italy, Hungary, Germany, Australia | Review | III | *COL3A1 gene, ARHGAP15, FAM155A and COLQ* |
| Stimac (41) | 31930228 | 2019 | Croatia, Italy | Review | III | *ARHGAP15, COLQ, FAM155A and CTAGE1* |
| Tursi (42) | 31204408 | 2019 | Italy | Review | III | *TNFSF15, LAMB4, COL3A1, ARHGAP15, COLQ, FAM155A, CTAGE1, NOV, PHGR1, CALCB and 100A10* |
| Weersma (43) | 30826747 | 2019 | UK | Review | III | Not specific |
| Winter (44) | 31350708 | 2019 | Ireland | Review | III | Not specific |
| Kadiyska (45) | 30809418 | 2018 | Bulgaria | Review | III | *TNFSF15* |
| Rezapour (46) | 28494576 | 2018 | USA | Review | III | Not specific |
| Violi (47) | 30561403 | 2018 | Italy | Review | III | Not specific |
| Tursi (48) | 26929783 | 2016 | Italy | Review | III | *TNFSF15* |
| Reichert (49) | 26535118 | 2015 | Germany | Review | III | *COL3A1, CLIP2, ELN, GTF2I, LIMK1, PKD1, PKD2, PKD3, RPS6KA3, TNFSF15, RPRM* |
| Tanase (50) | 25800310 | 2015 | USA | Systematic Review | III | Not specific |
| Templeton (51) | 24010157 | 2013 | USA | Review | III | Not specific |
| Commane (52) | 19468998 | 2009 | UK | Review | III | *MMP1, MMP2, TIMP1, COL5A1 or COL5A2, PKD1 or PKD2* |
| Petruzziello (53) | 16669953 | 2006 | Italy | Review | III | Not specific |
| Stumpf (54) | 17080262 | 2006 | Germany | Review | III | Not specific |

**S6** DNA/mRNA mutations associated with Diverticulitis/ Diverticulosis/ Diverticular Disease.

| **Functional Pathway** | **Disease Association** | **Gene** | **Chromosome** | **Position** | **Lead Variation** | **Sensitivity** | **Phenotype** |
| --- | --- | --- | --- | --- | --- | --- | --- |
| Altered Vasculature | Diverticulitis | *WNT4* | 1 | 22502000, 22531206, 22538787 | rs11799918, rs75637000, rs2473253 |  | Encode secreted signalling proteins implicated in oncogenesis and processes including regulation of cell fate and patterning during embryogenesis |
| Altered Vasculature | Diverticulitis, DD | *SLC35F3* | 1 | 234217153 | rs4333882 | P value: DD=4.44 × 10−22, Diverticulosis=0.0007, Diverticulitis=0.14 | Involved in thiamine transport predicted to be integral component of membrane |
| Altered Vasculature | DD, Diverticulosis | *ABO* | 9 | 136145484 | rs582094 | P value: DD=1.55 × 10−11, Diverticulosis=0.12, Diverticulitis=0.0008 | Encodes a glycosyltransferase that catalyzes the transfer of carbohydrates to the H antigen, forming the antigenic structures of the ABO blood group |
| Altered Vasculature | DD, Diverticulosis | *BMPR1B* | 4 | 94852434 | rs1544387 | P value: DD=5.74 × 10−9, Diverticulosis=0.023, Diverticulitis=0.0005 | Bone morphogenetic protein (BMP) receptors are a family of transmembrane serine/threonine kinases, the ligands of these receptors are members of the TGF-beta superfamily. |
| Altered Vasculature | DD | *P2RY12 (P2RY14)* | 3 | 151360428 | rs9856118 | P value: DD=8.80 × 10−11, Diverticulosis=0.15, Diverticulitis=0.82 | Encodes G-protein coupled receptors involved in platelet aggregation |
| Altered Vasculature | DD | *RHOU* | 1 | 228867648, 228878669, 228880135, 228880466 | rs72751907, rs4993975, rs11583565, rs11580020 |  | Encodes a proteinthat can activate PAK1 and JNK1, and can induce filopodium formation and stress fiber dissolution |
| Extracellular Matrix | Diverticulosis | *TIMP- 1* | x |  | rs4898 |  | Encodes natural inhibitors of the matrix metalloproteinases |
| Extracellular Matrix | Diverticulosis | *TIMP- 3* | 22 |  |  |  | Encodes natural inhibitors of the matrix metalloproteinases |
| Extracellular Matrix | Diverticulitis, DD | *C1QTNF7* | 4 | 15386383 | rs4132788 |  | Protein coding gene enables identical protein binding activity in the extracellular space. Part of collagen trimer. |
| Extracellular Matrix | Diverticulitis, DD | *TIMP- 2* | 17 | 76856966 | rs1973232 |  | Encodes natural inhibitors of the matrix metalloproteinases |
| Extracellular Matrix | Diverticulitis | *COL3A1* | 2 |  | rs3134646 |  | Encodes the pro-alpha1 chains of type III collagen |
| Extracellular Matrix | DD | *VTN* | 17 |  |  |  | Encode adhesive glycoproteins |
| Extracellular Matrix | Diverticulosis | *MMP-1* | 11 |  |  |  | Encodes a member of the peptidase M10 family of matrix metalloproteinase |
| Extracellular Matrix | DD | *HAS2* | 8 | 121246834 | rs4871180 | P value: DD=4.15 × 10−9, Diverticulosis=0.77, Diverticulitis=0.77 | Encodes hyaluronic acid (HA) |
| Extracellular Matrix | DD | *NOV (CCN3)* | 8 | 119415408 | rs60869342, rs1381335 | P value: DD=1.91 × 10−10, Diverticulosis=0.69, Diverticulitis=0.64 | Encodes a small secreted cysteine-rich protein and a member of the CCN family of regulatory proteins |
| Extracellular Matrix | DD | *SMMHC (MYH11)* | 16 |  |  |  | Encodes a smooth muscle myosin belonging to the myosin heavy chain family |
| Extracellular Matrix, Immune Function | Diverticulosis | *LAMB4* | 7 |  | rs2074749, rs2240445, rs147992634, rs149874137, rs9690688, and rs1627354 |  | Encodes part of the laminin complex an extracellular matrix structural constituent |
| Extracellular Matrix, Immune Function | Diverticulosis | *TNFSF15* | 9 |  | rs7848647 |  | Encodes a protein that is abundantly expressed in endothelial cells, but is not expressed in either B or T cells, this cytokine is a ligand for receptor TNFRSF25 and decoy receptor TNFRSF21/DR6 |
| Extracellular Matrix, Neuromuscular Function | DD | *COLQ6A1* | 21 | 45999606 | rs75434097 | P value: DD=4.90 × 10−11, Diverticulosis=0.42, Diverticulitis=0.98 | Encodes protein alpha 1 subunit of type VI collagen |
| Extracellular Matrix, Neuromuscular Function, Immune Function | DD, Diverticulosis | *COLQ* | 3 | 15461174 | rs7609897 | P value: DD=2.72 × 10−18, Diverticulosis=0.10, Diverticulitis=0.0096 (varient rs7609897-T: P value for DD=1.5 × 10-10, odds ratio=0.87) | Encodes a collagen-like strand that associates into a triple helix to form a tail that anchors catalytic subunits of acetylcholinesterase to the basal lamina. |
| Extracellular Matrix, Vascular Alteration | Diverticulitis, DD | *ELN (L1MK1)* | 7 | 74028915 | rs3823878 | P value: DD=2.63 × 10−9, Diverticulosis=0.0018, Diverticulitis=0.78 | Encodes a protein that is one of the two components of elastic fibers (associated with Aortic Stenosis and Cutis Laxida) |
| Extracellular Matrix, Vascular Alteration | DD | *EFEMP1* | 2 | 55866069 | rs1802575 | P value: DD=7.71 × 10−16, Diverticulosis=0.08, Diverticular Disease=0.07 | Encodes a member of the fibulin family of extracellular matrix glycoproteins (associated with malignant gliomas) |
| Immune Function | Diverticulosis | *IFIT1* | 10 |  |  |  | Encodes a protein containing tetratricopeptide repeats to inhibit viral replication and translational initiation |
| Immune Function | Diverticulosis | *INPP5D (SHIP1)* | 2 |  |  |  | Encodes a protein that functions as a negative regulator of myeloid cell proliferation and survival |
| Immune Function | Diverticulosis | *PTPRC* | 15 |  |  |  | Protein tyrosine phosphatase family known to be signaling molecules that regulate a variety of cellular processes including cell growth, differentiation, mitosis, and oncogenic transformation |
| Immune Function | Diverticulosis | *RASAL3* | 19 |  |  |  | Encodes a protein with pleckstrin homology (PH), C2, and Ras GTPase-activation protein (RasGAP) domains |
| Immune Function | Diverticulosis | *S100A10* | 8 | 151998153 | rs61814883 | P value: DD=2.05 × 10−10, Diverticulosis=0.46, Diverticulitis=0.60 Odds ratio:1.17 (95% CI 1.03 to 1.33) | Encodes the S100 family of proteins containing 2 EF-hand calcium-binding motifs |
| Immune Function | Diverticulosis | *SASH3* | x |  |  |  | Encodes a protein containing a Src homology-3 (SH3) domain and a sterile alpha motif (SAM), both of which are found in proteins involved in cell signaling |
| Immune Function | Diverticulitis, DD, Diverticulosis | *FAM155A (NALF1)* | 13 | 107250422, 107566610 | rs67153654, rs9520344 (LM), rs11619840 (LM) | Odd ratio: Divertuculitis 1.21, (95% CI 1.04 to 1.42) (varient rs9520344: P value: DD=5.23 × 10−12, Divertiuclosis 0.08, Diverticulitis=0.34) (varient rs11619840: DD=1.70 × 10−9, Diverticulosis=0.0042, Diverticulitis=0.021) (varient rs67153654-A: P value: Diverticulitis=3.0 × 10-11, Odd ratio: 0.82) | Contributes to stretch-activated, cation-selective, calcium channel activity in plasma membrane |
| Immune Function | Diverticulitis, DD | *ARHGAP15* | 2 | 143556678 (LM) | rs4662344, rs6734367 (LM) | P value: DD=4.29 × 10−44, Diverticulosis=0.0038, Diverticulitis=0.07 (varient rs4662344-T P value: DD=1.9 × 10-18, Odds ratio=1.23) | Regulates RHO GTPase-activating proteins (GAPs) |
| Immune Function | Diverticulitis, DD | *HLA- DQA1* | 6 | 32609965 | rs7990 |  | Encodes Major Histocompatibility Complex, Class II, DR Beta 1 |
| Immune Function | Diverticulitis | *OAS1/3* | 12 | 113365621, 113409176 | rs11066453, rs2072134 |  | Encodes a protein that synthesizes 2',5'-oligoadenylates, a key role in innate cellular antiviral response |
| Immune Function | DD | *CCL18* | 17 |  |  |  | Cys-Cys (CC) cytokine genes secreting proteins involved in immunoregulatory and inflammatory processes |
| Immune Function | DD | *CCL20* | 2 |  |  |  | Cys-Cys (CC) cytokine genes secreting proteins involved in immunoregulatory and inflammatory processes |
| Immune Function | DD | *CCL23* | 17 |  |  |  | Cys-Cys (CC) cytokine genes secreting proteins involved in immunoregulatory and inflammatory processes |
| Neuromuscular Alteration | Diverticulosis | *CACNA1G* | 17 |  |  |  | Encodes a subunit of a voltage-dependent calcium channel protein |
| Neuromuscular Alteration | DD | *CACNB2* | 10 | 18151515 | rs1888693 | P value: DD=3.58 × 10−9,Diverticlosis=0.10, Diverticulitis=0.50 | Encodes the low-voltage-activated Ca T-type calcium channel (associated with Lambert- Eaton Syndrome and Brugada Syndorme) |
| Neuromuscular Function | Diverticulosis | *CALCA* | 11 | 14993308 | rs12293535 | P value: DD=6.20 × 10−10 , Diverticulosis=0.37, Diverticulitis=0.47 | Encodes the peptide hormones calcitonin, vasodilator. |
| Neuromuscular Function | Diverticulosis | *SERT (SLC6A4)* | 17 |  |  |  | Encodes an integral membrane protein that transports the neurotransmitter serotonin from synaptic spaces into presynaptic neurons |
| Neuromuscular Function | Diverticulosis | *SNAP- 25* | 20 |  |  |  | Encodes synaptic vesicle membrane, docking and fusion is mediated by SNARE to the target membrane |
| Neuromuscular Function | Diverticulosis | *TACR1* | 17 |  |  |  | Encodes the receptor for the tachykinin substance P, also referred to as neurokinin 1 |
| Neuromuscular Function | Diverticulosis | *TACR2* | 10 |  |  |  | Encodes the receptor for the tachykinin neuropeptide substance K |
| Neuromuscular Function | Diverticulitis, DD | *PPP1R14A (CPI-17)* | 19 | 38425164 | rs11667256 | P value: DD=1.24 × 10−14, Diverticulosis=0.025, Diverticulitis=0.062 | Encodes the protein phosphatase 1 (PP1) inhibitor family an inhibitor of smooth muscle myosin phosphatase |
| Neuromuscular Function | DD | *ANO1* | 11 |  |  |  | Membrane proteins containing 8 transmembrane segments associated with calcium-activated chloride channel activity |
| Neuromuscular Function | DD | *GDNF* | 5 |  |  |  | Encodes a secreted ligand of the TGF-beta superfamily of proteins and regulates gene expression |
| Neuromuscular Function | DD | *GFRA1* | 10 |  |  |  | Encodes a member of the glial cell line-derived neurotrophic factor receptor family of proteins involved in receptor maturation |
| Neuromuscular Function | DD | *RET* | 10 |  |  |  | Eencodes a transmembrane receptor and member of the tyrosine protein kinase family of proteins, binds ligands such as GDNF that play a role in cell differentiation, growth, migration and survival |
| Neuromuscular Function, Vascular Alteration, Immune Function | Diverticulosis | *CALCB* | 11 |  |  | Odds ratio: Diverticulosis=1.17 (95% CI 1.03 to 1.33) | Enable calcitonin receptor binding activity in the extracellular space |
| Unknown | Diverticulosis | *ISG15* | 1 |  |  |  | Encodes a ubiquitin-like protein that is conjugated to intracellular target proteins upon activation by interferon-alpha and interferon-beta |
| Unknown | Diverticulitis, DD | *COX-2 (PTGS2)* | 1 |  |  |  | Encodes cyclooxygenase the key enzyme in prostaglandin biosynthesis (associated with gastric ulcers) |
| Unknown | Diverticulitis, DD | *FADD* | 11 | 70159268, 70247466 | rs875107, rs72945112 | (varient rs875107: P value: DD=2.33 × 10−9, Diverticulosis=0.0004, Diverticulitis=0.10) (variant rs72945112: P value: DD=6.30 × 10−6, Diverticulosis=0.0016, Diverticulitis=0.80) | Encodes an adaptor molecule that interacts with various cell surface receptors and mediates cell apoptotic signals |
| Unknown | Diverticulitis, DD | *GTPBP1* | 22 | 38733703 | rs1386991,2 | P value: DD=8.02 × 10−7, Diverticulosis=0.0039, Diverticulitis=0.25 | Encodes a protein that is a member of the AGP11/GTPBP1 family of GTP-binding proteins |
| Unknown | Diverticulitis, DD | *ISL2 (ETFA/ SCARPER)* | 15 | rs2056544, 76533662, 76286749 | rs2056544, rs10519134 | (varient rs2056544: P value: DD=1.01 × 10−7, Diverticulosis=0.99, Diverticulitis=0.08) (varient rs10519134: P value: DD=7.12 × 10−6, Diverticulosis=0.0036, Diverticulitis=0.66) | Enables sequence-specific double-stranded DNA binding activity |
| Unknown | Diverticulitis, DD | *PPP1R16B* | 20 | 37493576 | rs208814 |  | Encodes a membrane associated protein |
| Unknown | Diverticulitis, DD | *SHFM1 (CLSTN2)* | 7 | 96449252 | rs43113037 | P value: DD=2.52 × 10−10,Diverticulosis=0.0039, Diverticulitis=0.0033 | Enable calcium ion binding activity |
| Unknown | Diverticulitis | *BDNF* | 11 | 27712873 | rs962369 | P value: DD=2.16 × 10−14,Diverticulosis=0.18, Diverticulitis=0.74 | Neurotrophin acting at NGFR receptors |
| Unknown | Diverticulitis | *NT5C1B* | 2 | 18806974 | rs62126581 | P value: DD=3.77 × 10−8, Diverticulosis=0.62, Diverticulitis=0.23 | Encodes a cytosolic 5-prime nucleotidases that catalyze production of adenosine |
| Unknown | Diverticulitis | *PHOX2B* | 4 |  |  |  | Encodes a proteinthat functions as a transcription factor involved in the development of several major noradrenergic neuron populations and the determination of neurotransmitter phenotype |
| Unknown | DD, Diverticulosis | *GPR158* | 10 | 25522506 | rs7086249 | P value: DD=5.37 × 10−16, Diverticulosis=0.014, Diverticulitis=0.0004 | Enable G protein-coupled receptor activity in plasma membrane |
| Unknown | DD | *5HT-4R* | 5 |  |  |  | Encodes serotonin receptors |
| Unknown | DD | *CRISPLD2* | 16 | 84823772 | rs2131755 | P value: DD+1.50 × 10−10, Diverticulosis=0.06, Diverticulitis=0.59 | Enable glycosaminoglycan binding activity (associated with cleft lip) |
| Unknown | DD | *CTAGE1* | 18 | 20028737 | rs9960286 | P value: DD=2.3×10-10 and 0.002 Odds ratio=1.14 (95% CI 1.05 to 1.24) | Protein coding gene enabling endoplasmic reticulum to Golgi vesicle-mediated transport, protein secretion and vesicle cargo loading (associated with Meckels diverticulum and Lymphoma) |
| Unknown | DD | *CUTC* | 12 |  |  |  | Copper transporters are associated with copper homeostasis |
| Unknown | DD | *CWC27* | 5 | 64999536 | rs10471645 | P value: DD=3.03 × 10−9, Diverticulosis=0.037, Diverticulitis=0.0091 | Enable peptidyl-prolyl cis-trans isomerase activity (associated with retinal pigmentosa) |
| Unknown | DD | *DISP2* | 15 | 40357408 | rs71472433 | P value: DD=8.90 × 10−11, Diverticulosis=0.74, Diverticulitis=0.89 | Related pathways are Regulation of activated PAK-2p34 by proteasome mediated degradation and Signaling by Hedgehog during embryonic pattern formation |
| Unknown | DD | *EDEM1* | 3 | 5804815 | rs2470653 | P value: DD=4.51 × 10−8, Diverticulosis=1.00, Diverticulitis=0.20 | Enables mannosyl-oligosaccharide 1,2-alpha-mannosidase activity and misfolded protein binding activity (associated with long QT and hepatocellular carcinoma) |
| Unknown | DD | *ENSG00000226849* | 6 | 98612512 | rs4839715 | P value: DD=1.62 × 10−10, Diverticulosis=0.87, Diverticulitis=0.26 | RNA Gene, and is affiliated with the lncRNA class |
| Unknown | DD | *ENSG00000251283 (LINC02272)* | 4 | 156636431 | rs11934833 | P value: DD=6.21 × 10−9, Diverticulosis=0.0053, Diverticulitis=0.30 | RNA Gene, and is affiliated with the lncRNA class |
| Unknown | DD | *FAM185A (FBXL13)* | 7 | 102806416 | rs6949391 | P value: DD=3.74 × 10−14, Diverticulosis=0.17, Diverticulitis=0.45 | Unknown |
| Unknown | DD | *HLX* | 1 | 220893031 | rs2784255 | P vaule: DD=1.06 × 10−8, Diverticulosis=0.99, Diverticulitis=0.61 | Enables sequence-specific DNA binding activity, involved in cell differentiation and regulation of transcription by RNA polymerase II |
| Unknown | DD | *LINC01082* | 16 | 86199807 | rs2280028 | P value: DD=7.05 × 10−11, Diverticulosis=0.73, Diverticulitis=0.10 | RNA Gene, and is affiliated with the lncRNA class |
| Unknown | DD | *LYPLAL1- AS1* | 1 | 219121228 | rs61823192 | P value: DD=1.15 × 10−13, Diverticulosis=0.16, Diverticulitis=0.021 | RNA Gene, and is affiliated with the lncRNA class |
| Unknown | DD | *PCSK5* | 9 | 76125437 | rs10120333 | P value: DD=1.54 × 10−8, Diverticulosis=0.92, Diverticulitis=0.39 | Encodes a member of the subtilisin-like proprotein convertase family, which includes proteases that process protein and peptide precursors trafficking through regulated or constitutive branches of the secretory pathway |
| Unknown | DD | *PIAS1* | 15 | 68238462 | rs387505 |  | Encodes a member of the protein inhibitor of activated STAT (PIAS) family |
| Unknown | DD | *RBKS* | 2 | 28065525 | rs10173528 | P value: DD=4.73 × 10−8, Diverticulosis=0.82, Diverticulitis=0.94 | Encodes the protein phosphorylates ribose to form ribose-5-phosphate in the presence of ATP and magnesium as a first step in ribose metabolism |
| Unknown | DD | *RPRM* | 2 |  |  |  | Encodes a protein involved in the regulation of mitotic cell cycle |
| Unknown | DD | *SLC25A28* | 10 | 99631412 | rs7098322 | P value: DD=9.94 × 10−12, Diverticulosis=0.67, Diverticulitis=0.33 | Enable ferrous iron transmembrane transporter activity |
| Unknown | DD | *SLC4A1* | 17 |  |  |  | Encodes proteins in the anion exchanger family and is expressed in the erythrocyte plasma membrane, where it functions as a chloride/bicarbonate exchanger involved in carbon dioxide transport |
| Unknown | DD | *SNX24* | 5 | 122329729 | rs34126945 |  | Enables phosphatidylinositol phosphate binding activity |
| Unknown | DD | *SPINT2* | 19 |  |  |  | Encodes a transmembrane protein with two extracellular Kunitz domains that inhibits a variety of serine proteases. The protein inhibits HGF activator which prevents the formation of active hepatocyte growth factor. This gene is a putative tumor suppressor, and mutations in this gene result in congenital sodium diarrhea |
| Unknown | DD | *TNRC6B* | 22 | 40695172 | rs6001870 |  | Enables RNA binding activity |
| Unknown | DD | *TRPS1* | 8 | 115576319 | rs2049865 | P value: DD=5.54 × 10−9, Diverticulosis=0.59, Diverticulitis=0.52 | Encodes a transcription factor that represses GATA-regulated genes and binds to a dynein light chain protein |
| Unknown | DD | *UBL4B* | 1 | 110120397 | rs115490395 | P value: DD=4.39 × 10−8, Diverticulosis=0.42, Diverticulitis=0.81 | Enables positive regulation of protein targeting to mitochondrion |
| Unknown | DD | *UBTF* | 17 | 44235410 | rs8074740 | P value: DD=2.34 × 10−10, Diverticulosis=0.88, Diverticulitis=0.08 | Encodes a member of the HMG-box DNA-binding protein family and plays a critical role in ribosomal RNA transcription |
| Unknown | DD | *UNC50* | 2 | 98612512 | rs148376933 | P value: DD=1.88 × 10−10, Diverticulosis=0.91, Diverticulitis=0.49 | Enable RNA binding activity |
| Unknown | DD | *WDR70* | 5 | 37772678 | rs10372291 | P value: DD=1.01 × 10−11, Diverticulosis=0.21, Diverticulitis=0.89 | Enables enzyme binding activity |
| Unknown | DD | *ZBTB4* | 17 | 7469318 | rs12942267 | P value: DD=2.55 × 10−8, Diverticulosis=0.042, Diverticulitis=0.73 | Enables functions, including DNA-binding transcription repressor activity, RNA polymerase II-specific; methyl-CpNpG binding activity; and sequence-specific DNA binding activity |

**References**

1. Joo YY, Pacheco JA, Thompson WK, Rasmussen-Torvik LJ, Rasmussen LV, Lin FTJ, et al. Multi-ancestry genome- and phenome-wide association studies of diverticular disease in electronic health records with natural language processing enriched phenotyping algorithm. PLoS One. 2023;18(5):e0283553.

2. Choe EK, Lee JE, Chung SJ, Yang SY, Kim YS, Shin ES, et al. Genome-wide association study of right-sided colonic diverticulosis in a Korean population. Sci Rep. 2019;9(1):7360.

3. Schafmayer C, Harrison JW, Buch S, Lange C, Reichert MC, Hofer P, et al. Genome-wide association analysis of diverticular disease points towards neuromuscular, connective tissue and epithelial pathomechanisms. Gut. 2019;68(5):854-65.

4. Maguire LH, Handelman SK, Du X, Chen Y, Pers TH, Speliotes EK. Genome-wide association analyses identify 39 new susceptibility loci for diverticular disease. Nat Genet. 2018;50(10):1359-65.

5. Sigurdsson S, Alexandersson KF, Sulem P, Feenstra B, Gudmundsdottir S, Halldorsson GH, et al. Sequence variants in ARHGAP15, COLQ and FAM155A associate with diverticular disease and diverticulitis. Nature Communications. 2017;8:15789.

6. Noyes JD, Mordi IR, Doney AS, Palmer CNA, Pearson ER, Lang CC. Genetic Risk of Diverticular Disease Predicts Early Stoppage of Nicorandil. Clin Pharmacol Ther. 2020;108(6):1171-5.

7. Reichert MC, Kupcinskas J, Krawczyk M, Jungst C, Casper M, Grunhage F, et al. A variant of COL3A1 (rs3134646) is associated with risk of developing diverticulosis in white men. Diseases of the Colon and Rectum. 2018;61(5):604-11.

8. Schieffer KM, Kline BP, Harris LR, Deiling S, Koltun WA, Yochum GS. A differential host response to viral infection defines a subset of earlier-onset diverticulitis patients. J Gastrointestin Liver Dis. 2018;27(3):249-55.

9. Schieffer KM, Choi CS, Emrich S, Harris L, Deiling S, Karamchandani DM, et al. RNA-seq implicates deregulation of the immune system in the pathogenesis of diverticulitis. Am J Physiol Gastrointest Liver Physiol. 2017;313(3):G277-g84.

10. Reichert MC, Kupcinskas J, Schulz A, Schramm C, Weber SN, Krawczyk M, et al. Common variation in FAM155A is associated with diverticulitis but not diverticulosis. Sci Rep. 2020;10(1):1658.

11. Kline BP, Schieffer KM, Choi CS, Connelly T, Chen J, Harris L, et al. Multifocal Versus Conventional Unifocal Diverticulitis: A Comparison of Clinical and Transcriptomic Characteristics. Digestive Diseases & Sciences. 2019;64(11):3143-51.

12. Connelly TM, Choi CS, Berg AS, Harris L, Coble J, Koltun WA. Diverticulitis and Crohn's disease have distinct but overlapping tumor necrosis superfamily 15 haplotypes. Journal of Surgical Research. 2017;214:262-9.

13. Barrenschee M, Böttner M, Harde J, Lange C, Cossais F, Ebsen M, et al. SNAP-25 is abundantly expressed in enteric neuronal networks and upregulated by the neurotrophic factor GDNF. Histochem Cell Biol. 2015;143(6):611-23.

14. Dai L, King DW, Perera DS, Lubowski DZ, Burcher E, Liu L. Inverse expression of prostaglandin E2-related enzymes highlights differences between diverticulitis and inflammatory bowel disease. Dig Dis Sci. 2015;60(5):1236-46.

15. Connelly TM, Berg AS, Hegarty JP, Deiling S, Brinton D, Poritz LS, et al. The TNFSF15 gene single nucleotide polymorphism rs7848647 is associated with surgical diverticulitis. Ann Surg. 2014;259(6):1132-7.

16. Hellwig I, Böttner M, Barrenschee M, Harde J, Egberts JH, Becker T, et al. Alterations of the enteric smooth musculature in diverticular disease. J Gastroenterol. 2014;49(8):1241-52.

17. Böttner M, Barrenschee M, Hellwig I, Harde J, Egberts JH, Becker T, et al. The GDNF System Is Altered in Diverticular Disease - Implications for Pathogenesis. PLoS One. 2013;8(6):e66290.

18. von Rahden BH, Jurowich C, Kircher S, Lazariotou M, Jung M, Germer CT, et al. Allergic predisposition, histamine and histamine receptor expression (H1R, H2R) are associated with complicated courses of sigmoid diverticulitis. J Gastrointest Surg. 2012;16(1):173-82; discussion 82.

19. Liu L, Markus I, Saghire HE, Perera DS, King DW, Burcher E. Distinct differences in tachykinin gene expression in ulcerative colitis, Crohn's disease and diverticular disease: a role for hemokinin-1? Neurogastroenterol Motil. 2011;23(5):475-83, e179-80.

20. Costedio MM, Coates MD, Danielson AB, Buttolph TR, 3rd, Blaszyk HJ, Mawe GM, et al. Serotonin signaling in diverticular disease. J Gastrointest Surg. 2008;12(8):1439-45.

21. Rottier SJ, de Jonge J, Dreuning LC, van Pelt J, van Geloven AAW, Beele XDY, et al. Prevalence of alpha-1-antitrypsin deficiency carriers in a population with and without colonic diverticula. A multicentre prospective case-control study: the ALADDIN study. Int J Colorectal Dis. 2019;34(5):933-8.

22. Niessen P, Rensen S, van Deursen J, De Man J, De Laet A, Vanderwinden JM, et al. Smoothelin-a is essential for functional intestinal smooth muscle contractility in mice. Gastroenterology. 2005;129(5):1592-601.

23. Kline BP, Yochum GS, Brinton DL, Schieffer KM, Weaver T, Harris L, et al. COLQ and ARHGAP15 are associated with diverticular disease and are expressed in the colon. J Surg Res. 2021;267:397-403.

24. Cossais F, Lange C, Barrenschee M, Möding M, Ebsen M, Vogel I, et al. Altered enteric expression of the homeobox transcription factor Phox2b in patients with diverticular disease. United European Gastroenterol J. 2019;7(3):349-57.

25. Coble JL, Yue F, Salameh TJ, Harris LR, Deiling S, Ruggiero FM, et al. Identification of a rare LAMB4 variant associated with familial diverticulitis through exome sequencing. Human Molecular Genetics. 2017;26(16):3212-20.

26. Ha SE, Lee MY, Kurahashi M, Wei L, Jorgensen BG, Park C, et al. Transcriptome analysis of PDGFRalpha+ cells identifies T-type Ca2+ channel CACNA1G as a new pathological marker for PDGFRalpha+ cell hyperplasia. PLoS ONE. 2017;12(8):e0182265.

27. Connelly TM, Berg AS, Harris LR, Tappouni R, Brinton D, Deiling S, et al. Surgical diverticulitis is not associated with defects in the expression of wound healing genes. International Journal of Colorectal Disease. 2015;30(9):1247-54.

28. Strate LL, Erichsen R, Baron JA, Mortensen J, Pedersen JK, Riis AH, et al. Heritability and familial aggregation of diverticular disease: A population-based study of twins and siblings. Gastroenterology. 2013;144(4):736-42.e1.

29. Granlund J, Svensson T, Olen O, Hjern F, Pedersen NL, Magnusson PKE, et al. The genetic influence on diverticular disease - A twin study. Alimentary Pharmacology and Therapeutics. 2012;35(9):1103-7.

30. Saylors S, Schaeffer HD, Dove J, Haley J, Smelser D, Carey D, et al. Utilization of Genetically Inferred Pedigrees in a Large Clinical Population to Study Diverticulitis. J Gastrointest Surg. 2023.

31. Cohan JN, Horns JJ, Hanson HA, Allen-Brady K, Kieffer M, Huang LC, et al. The Association between Family History and Diverticulitis Recurrence: A Population-Based Study. Dis Colon Rectum. 2021.

32. Broad JB, Wu Z, Clark TG, Musson D, Jaung R, Arroll B, et al. Diverticulosis and nine connective tissue disorders: epidemiological support for an association. Connect Tissue Res. 2019;60(4):389-98.

33. Mastoraki A, Schizas D, Tousia A, Chatzopoulos G, Gkiala A, Syllaios A, et al. Evaluation of molecular and genetic predisposing parameters at diverticular disease of the colon. Int J Colorectal Dis. 2021;36(5):903-10.

34. Peery AF, Shaukat A, Strate LL. AGA Clinical Practice Update on Medical Management of Colonic Diverticulitis: Expert Review. Gastroenterology. 2021;160(3):906-11.e1.

35. Maguire LH. Genetic Risk Factors for Diverticular Disease-Emerging Evidence. J Gastrointest Surg. 2020;24(10):2314-7.

36. Miulescu AM. Colonic Diverticulosis. Is there a Genetic Component? Maedica (Bucur). 2020;15(1):105-10.

37. Nasef NA, Mehta S. Role of Inflammation in Pathophysiology of Colonic Disease: An Update. Int J Mol Sci. 2020;21(13).

38. Tursi A, Scarpignato C, Strate LL, Lanas A, Kruis W, Lahat A, et al. Colonic diverticular disease. Nat Rev Dis Primers. 2020;6(1):20.

39. Camilleri M, Sandler RS, Peery AF. Etiopathogenetic Mechanisms in Diverticular Disease of the Colon. Cell Mol Gastroenterol Hepatol. 2020;9(1):15-32.

40. Kupcinskas J, Strate LL, Bassotti G, Torti G, Herszènyi L, Malfertheiner P, et al. Pathogenesis of Diverticulosis and Diverticular Disease. J Gastrointestin Liver Dis. 2019;28(suppl. 4):7-10.

41. Stimac D, Nardone G, Mazzari A, Crucitti A, Maconi G, Elisei W, et al. What's new in diagnosing diverticular disease. Journal of Gastrointestinal and Liver Diseases. 2019;28(Supplement 4):17-21.

42. Tursi A. Current and Evolving Concepts on the Pathogenesis of Diverticular Disease. J Gastrointestin Liver Dis. 2019;28:225-35.

43. Weersma RK, Parkes M. Diverticular disease: picking pockets and population biobanks. Gut. 2019;68(5):769-70.

44. Winter DC. Concise Commentary: Pocketing the Difference-Genetics and the Changing Paradigms of Diverticulitis Management. Dig Dis Sci. 2019;64(11):3152-3.

45. Kadiyska T, Tourtourikov I, Popmihaylova AM, Kadian H, Chavoushian A. Role of TNFSF15 in the intestinal inflammatory response. World J Gastrointest Pathophysiol. 2018;9(4):73-8.

46. Rezapour M, Ali S, Stollman N. Diverticular Disease: An Update on Pathogenesis and Management. Gut Liver. 2018;12(2):125-32.

47. Violi A, Cambiè G, Miraglia C, Barchi A, Nouvenne A, Capasso M, et al. Epidemiology and risk factors for diverticular disease. Acta Biomed. 2018;89(9-s):107-12.

48. Tursi A. Diverticulosis today: unfashionable and still under-researched. Therap Adv Gastroenterol. 2016;9(2):213-28.

49. Reichert MC, Lammert F. The genetic epidemiology of diverticulosis and diverticular disease: Emerging evidence. United European Gastroenterol J. 2015;3(5):409-18.

50. Tănase I, Păun S, Stoica B, Negoi I, Gaspar B, Beuran M. Epidemiology of diverticular disease -- systematic review of the literature. Chirurgia (Bucur). 2015;110(1):9-14.

51. Templeton AW, Strate LL. Updates in diverticular disease. Curr Gastroenterol Rep. 2013;15(8):339.

52. Commane DM, Arasaradnam RP, Mills S, Mathers JC, Bradburn M. Diet, ageing and genetic factors in the pathogenesis of diverticular disease. World J Gastroenterol. 2009;15(20):2479-88.

53. Petruzziello L, Iacopini F, Bulajic M, Shah S, Costamagna G. Review article: uncomplicated diverticular disease of the colon. Aliment Pharmacol Ther. 2006;23(10):1379-91.

54. Stumpf M, Krones CJ, Klinge U, Rosch R, Junge K, Schumpelick V. Collagen in colon disease. Hernia. 2006;10(6):498-501.
